# Observation of conformational changes that underlie the catalytic cycle of Xrn2

**DOI:** 10.1038/s41589-022-01111-6

**Published:** 2022-08-25

**Authors:** Jan H. Overbeck, David Stelzig, Anna-Lisa Fuchs, Jan Philip Wurm, Remco Sprangers

**Affiliations:** 1 Institute of Biophysics and Physical Biochemistry, Regensburg Center for Biochemistry, University of Regensburg, Regensburg, Germany; 2 Present Address: Department of Informatics, TU Munich, Garching, Germany

**Keywords:** Enzymes, NMR spectroscopy, X-ray crystallography

## Abstract

Nuclear magnetic resonance (NMR) methods that quantitatively probe motions on molecular and atomic levels have propelled the understanding of biomolecular processes for which static structures cannot provide a satisfactory description. In this work, we studied the structure and dynamics of the essential 100-kDa eukaryotic 5′→3′ exoribonuclease Xrn2. A combination of complementary fluorine and methyl-TROSY NMR spectroscopy reveals that the apo enzyme is highly dynamic around the catalytic center. These observed dynamics are in agreement with a transition of the enzyme from the ground state into a catalytically competent state. We show that the conformational equilibrium in Xrn2 shifts substantially toward the active state in the presence of substrate and magnesium. Finally, our data reveal that the dynamics in Xrn2 correlate with the RNA degradation rate, as a mutation that attenuates motions also affects catalytic activity. In that light, our results stress the importance of studies that go beyond static structural information.

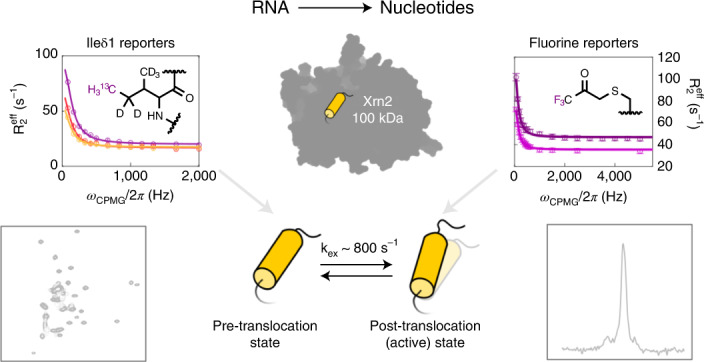

## Abstract (web summary)

Using methyl group and fluorine NMR spectroscopic methods, Overbeck et al revealed that the dynamics of the eukaryotic 5′→3′ exoribonuclease Xrn2 in the region around the active site are correlated with its catalytic activity.

## Main

5′→3′ exoribonucleases are essential enzymes that processively remove nucleotides from the 5′ end of RNA^1–4^. In the nucleus, this process is carried out by the exoribonuclease Xrn2 (Rat1), which is important for the removal of aberrant pre-mRNAs^5^, rRNA maturation and decay^6–9^, the processing of small nucleolar RNA (snoRNA)^10,11^, transcription termination^12,13^, telomere length regulation^14^ and the degradation of microRNAs^15^ and hypomodified transfer RNAs (tRNAs)^16^. This multitude of cellular functions renders Xrn2 essential in *Saccharomyces cerevisiae*^17,18^ and *Schizosaccharomyces pombe*^19,20^ and during development in *Caenorhabditis elegans*^21^.

The molecular basis of RNA degradation by Xrn2 and its cytoplasmic paralogue Xrn1 has been established by several studies. Both enzymes are highly selective for substrates with exposed 5′-monophosphorylated ends and require divalent cations to catalyze the hydrolysis of phosphodiester bonds^22,23^. Xrn enzymes are highly processive and remove tens of nucleotides per second from the substrate^24–28^. The combination of processivity and speed is important, as this enables Xrn1 to closely follow ribosomes in co-translational mRNA decay^29–32^ and allows Xrn2 to chase RNA polymerase II in co-transcriptional RNA degradation after cleavage of the pre-mRNA at the polyadenylation signal^33^.

**Fig. 1:**
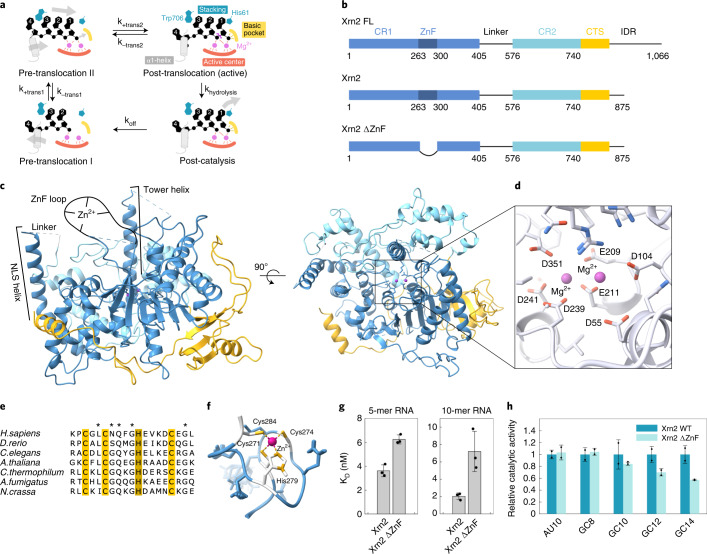
Structure of the Xrn2 enzyme from the thermophilic eukaryote *C. thermophilum*. **a**, The Xrn1/Xrn2 enzyme sandwiches the first three bases of the substrate RNA between a Trp and a His residue (top left; pre-translocation state II). Subsequently, the enzyme undergoes a conformational change and adopts the active conformation (top right). After hydrolysis (bottom right) and product release (bottom left), the substrate moves one base farther (top left). **b**, Schematic representation of the domain architecture of the full-length Xrn2 protein (top) and two constructs (Xrn2: middle; Xrn2 ΔZnF: bottom) used in this study. Solid boxes represent folded regions, whereas the linker region between CR1 and CR2 as well as the C-terminal intrinsically disordered region (IDR) are predicted to be unstructured and represented as lines. **c**, Crystal structure of CtXrn2, color-coded according to **b**. CR1 and CR2 and the Xrn2-specific CTS form a globular Xrn-core, in which the active site is accessible only from the top. **d**, Close-up of the active site, where seven conserved acidic residues (shown as sticks) coordinate two Mg^2+^ ions. **e**, Sequence alignment of the Xrn2 ZnF motif. The residues that coordinate the Zn^2+^ ion are highlighted in yellow (cysteines) and brown (histidine); other conserved residues are indicated with an asterisk. **f**, NMR structure of the ZnF region. **g**, Affinities of 5-mer and 10-mer RNA to WT Xrn2 and Xrn2 ΔZnF. Note that the binding experiments were performed in the absence of Mg^2+^ and in the presence of Zn^2+^. Data are shown as mean ± s.d. and were performed as triplicates. **h**, Relative degradation rates of Xrn2 WT and Xrn2 ΔZnF for different RNAs (Supplementary Table 4) containing either a 10-nucleotide AU hairpin (AU10) or GC hairpins with 8, 10, 12 or 14 nucleotides (GC8, GC10, GC12 and GC14). Data are shown as mean ± s.d. and were performed as two biological duplicates. Source data

On a structural level, Xrn1 and Xrn2 share two conserved regions (CR1 and CR2) and a less conserved C-terminal segment (CTS), which together form the Xrn core domain^25,27,34^ (Fig. 1a–c and Supplementary Figs. 1 and 2). This Xrn core harbors the catalytic site, which has three crucial features. First: a set of seven conserved acidic residues that mediate the coordination of two divalent cations, which are important for catalysis and substrate binding^25–27,35,36^ (Fig. 1d). Second: an adjacent basic binding pocket that is responsible for the selective recognition of the 5′ phosphate of the substrate^27^. This pocket rationalizes that neither unphosphorylated (no recognition) nor 5′-triphosphorylated or 5′-capped RNAs (steric hindrance) are substrates of Xrn1 or Xrn2. Third: two conserved residues (one histidine and one tryptophan) that sandwich the aromatic rings of the first three substrate nucleotides in an extensive base stack, thereby contributing to the processivity of the enzyme^27^. This static view of the enzyme:substrate complex has been expanded by the comparison of two Xrn1 structures that display different states during the catalytic cycle (Fig. 1a and Supplementary Fig. 2). First: a crystal structure of the *Drosophila melanogaster* Xrn1 enzyme in complex with a pseudo-substrate DNA displays the enzyme in a post-translocation, pre-hydrolysis (active) state. This state was stabilized by a mutation that disturbed one of the Mg^2+^ binding sites as well as by the missing 2′-hydroxyl group in the substrate^27^. A second snapshot of the catalytic cycle was provided by a cryogenic electron microscopy (cryo-EM) structure of *S. cerevisiae* Xrn1 that is stably bound to a stalled ribosome and in the presence of an mRNA substrate. In this latter structure, the π–π stack of the first three bases could be observed, but, at the same time, the terminal 5′-phosphate had yet to be fully translocated to the basic pocket^30^. This state was interpreted as a pre-translocation state. Based on these snapshots, it was noted that substantial conformational changes accompany the substrate translocation step, with a particularly pronounced rearrangement taking place at the N-terminal α1-helix of Xrn1 (ref. ^30^). This helix is functionally important, and it has been postulated to constitute a steric hindrance for double-stranded RNA^27^. In addition, deletion of the first four residues results in reduced catalytic activity^26^. Although no structural data have been reported for Xrn2 in a substrate-bound state, the conservation of CR1 and CR2 between Xrn1 and Xrn2 indicate that both enzymes share a common molecular mechanism, in which the N-terminal α1-helix can adopt two functionally important conformations.

In this work, we studied the interplay of structure and dynamics in the 100-kDa Xrn2 enzyme. To that end, we combined X-ray crystallography with NMR relaxation dispersion measurements on ^13^CH_3_-labeled and ^19^F-labeled Xrn2 samples. We found that the N-terminal region of the apo enzyme transiently populates an excited state that resembles the pre-hydrolysis substrate-bound state. The functional relevance of the observed helix dynamics is emphasized by a mutant enzyme in which motions and catalytic activity of Xrn2 are reduced.

## Results

### *Chaetomium thermophilum* Xrn2 is a canonical 5′→3′ exoribonuclease

High-resolution NMR studies that address protein dynamics require isotope-labeled samples that are stable over multiple days at protein concentration in the high μM range. Obtaining such samples is often a bottleneck, especially for large eukaryotic enzymes. Here, we found that the Xrn2 core from the thermophilic yeast *Chaetomium thermophilum* (CtXrn2, residues 1–875, molecular weight 100 kDa; Fig. 1b and Supplementary Table 1) fulfills those conditions. To lay the structural foundation for our NMR experiments, we first determined the crystal structure of CtXrn2 (Fig. 1c and Supplementary Table 7). In agreement with the high sequence conservation, we found that the CtXrn2 structure closely resembles those of *S. pombe*^25^ and *C. elegans*^37^. In addition, the CtXrn2 structure is similar to the core structures of the Xrn1 paralogues from *D. melanogaster*^27^, *Kluyveromyces lactis*^26^ and *S. cerevisiae*^30^ (Supplementary Fig. 3).

The active site of the enzyme is positioned in a pocket in front of the ‘tower’ domain helix (helix α4) (Fig. 1c). Seven acidic residues provide a coordination platform for the two catalytically important Mg^2+^ ions (Fig. 1d). Furthermore, we found that His61 and Trp706 are correctly positioned above the active site to form a continuous π–π stack with the RNA substrate, as has been observed for Xrn1 in complex with a pseudo-substrate DNA^27^ and an mRNA substrate in the context of a stalled ribosome^30^ (Supplementary Fig. 4).

### *C. thermophilum* Xrn2 contains a functional zinc finger

**Extended Data Fig. 1:**
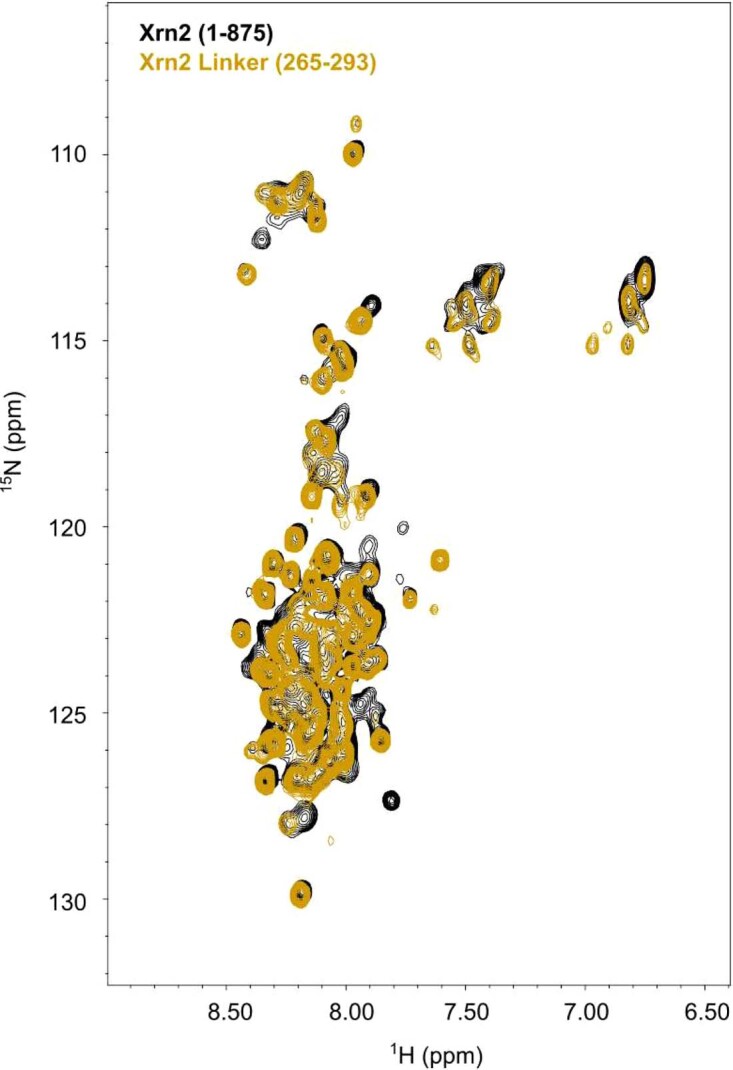
^1^H-^15^N TROSY spectra of Xrn2 (residues 1-875) and the Xrn2 linker (residues 265-293) that connects CR1 and CR2. The ^1^H-^15^N TROSY NMR spectrum of Xrn2 (black; residues 1-875) displays only ^1^H-^15^N correlations from highly flexible parts of the protein; the ^1^H-^15^N resonances in the protein core are broadened beyond detection due to the high molecular weight of the enzyme. The ^1^H-^15^N spectrum from the isolated Xrn2 linker region (yellow; residues 265-293) largely overlaps with the spectrum from Xrn2 (1-875), proving that the CR1-CR2 linker region is flexible and disordered in the context of the full length protein.

**Extended Data Fig. 2:**
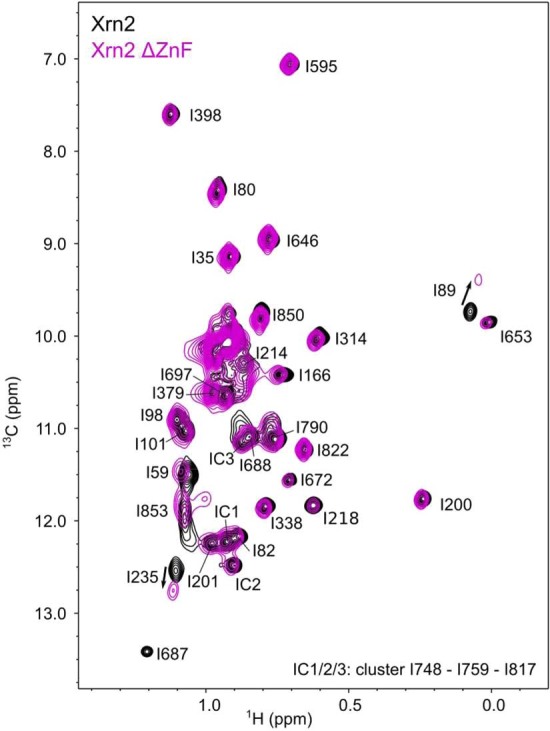
The ZnF transiently interacts with the Xrn2 core. Overlay of methyl TROSY NMR spectra of the Xrn2 enzyme with (black, WT) and without (purple, Delta ZnF) the ZnF. Removal of the ZnF does not perturb the structure of the Xrn2 enzyme, as can be concluded from the limited number of residues that experience CSPs. The observed CSPs upon deletion of the ZnF can be explained by the proximity of these residues to the (deleted) ZnF (for example I235) and by a transient interactions between the ZnF and a region of the enzyme that is located around the active site (I89, I59, I166, I314 and I850). In summary, these data thus show that the ZnF is loosely associated with Xrn2 and transiently interacts with the region of the enzyme that also contains the active site.

In our structure of the Xrn2 enzyme, electron density is lacking for three regions: (1) the top of the tower domain (residues 135–158) that is also less well defined in other Xrn2 structures; (2) the linker region between CR1 and CR2 (residues 423–568) that is highly flexible in solution (Extended Data Fig. 1); and (3) a short loop between residues 266 and 304 that harbors a putative CCHC zinc finger (ZnF) motif. Such a CX_2_CX_4_HX_4_C ZnF motif (where C, H and X correspond to a cysteine, a histidine and any amino acid, respectively) is present in Xrn2 homologues from higher eukaryotes (Fig. 1e), including *C. elegans*, where it was deleted from the crystallization construct^37^, and in a limited number of yeast species (for example, *Neurospora crassa* and *Aspergillus fumigatus*). However, the ZnF is missing in Xrn2 homologues from other Ascomycota (inluding *S. pombe*^25^ and *S. cerevisiae*) and is also generally absent in Xrn1. To obtain insights into the functional importance of the Xrn2 ZnF, we solved the NMR structure of this region (Fig. 1f). The structural ensemble (Supplementary Fig. 5 and Supplementary Table 8) displays a well-defined core (residues 269–284) that is similar to ZnFs that interact with (deoxy-)ribonucleotides (Supplementary Fig. 6). In the context of the full-length protein, the ZnF is localized on the side of the enzyme that also contains the active site (Fig. 1c and Extended Data Fig. 2), which prompted us to determine if the Xrn2 ZnF is important for substrate recruitment and/or turnover. To test this, we first measured the binding affinity of 5-mer and 10-mer RNAs to Xrn2. These experiments were recorded in the absence of Mg^2+^ to prevent the rapid degradation of the RNA substrate, and we found that the substrates bind with low nanomolar affinities (Fig. 1g). We next ‘inactivated’ the ZnF by using an Xrn2 ΔZnF construct. This increased the dissociation constant (reduced the affinity) by a factor of 1.5 (5-mer) to 3 (10-mer), corresponding to a ∆G < 2.7 kilojoules per mole (kJ/mol) at room temperature (Fig. 1g). Second, we investigated if the ZnF plays a role in Xrn2 activity (Supplementary Fig. 7). To that end, we compared the degradation rates of the Xrn2 wild-type (WT) protein with a version of the enzyme that lacks the ZnF. As the importance of the ZnF might depend on the extent of secondary structure in the substrate, we performed degradation assays using five RNA substrates with different stem–loop stabilities (Fig. 1h). We found that RNAs with a stem–loop of low stability were degraded with similar rates in the presence or absence of the ZnF. For RNAs that contain increasingly stable (GC) stem–loop elements, the deletion of the ZnF decreased the relative degradation rate accordingly. Based on the above, we conclude that the ZnF in Xrn2 plays a functionally important role in RNA binding and that it promotes the degradation of structured RNA substrates.

### Free Xrn2 displays dynamics in solution

Static structures from the Xrn2 paralogue Xrn1 indicate that the N-terminal α1-helix adopts different conformations in the apo and substrate-bound states and that the associated motions are correlated with translocation of the RNA substrate toward the active site (Fig. 1a and Supplementary Fig. 2)^27,30^. The sequence and structure of the α1-helices from Xrn2 and Xrn1 are conserved (Supplementary Fig. 8), although it should be noted that the electron density for the first residues is less well defined in the Xrn1 and Xrn2 crystal structures in the apo state^25,26,37^. Although there are no direct structural data for substrate-bound Xrn2, we reasoned that Xrn2 and Xrn1 undergo similar conformational changes during the catalytic cycle. In this process, the N-terminal α1-helix has been suggested to function as a Brownian ratchet that supports the translocation of the substrate RNA (Fig. 1a and Supplementary Fig. 2)^27^.

**Fig. 2:**
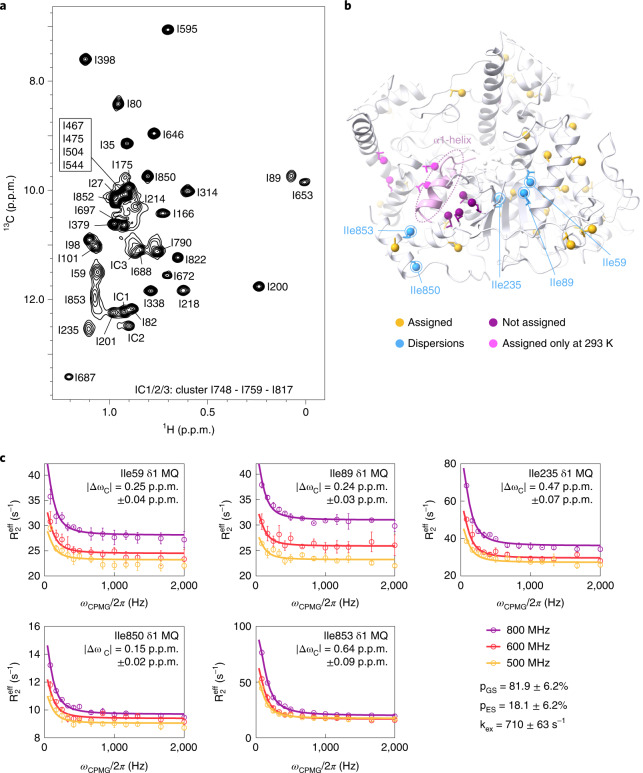
Methyl-TROSY NMR reveals conformational dynamics located around the N-terminal α1-helix. **a**, Methyl-TROSY spectrum of U-^2^H, Ile-δ_1_-labeled Xrn2 recorded at 800 MHz ^1^H frequency and 313 K with assignments (Supplementary Methods and Supplementary Table 2). Isoleucines I467, I475, I504 and I544 are located in the flexible linker between CR1 and CR2 and were assigned by comparison of HMQC spectra from the Xrn2 WT protein and an Xrn2 ∆Linker construct. Isoleucines I748, I759 and I817 are in close spatial contact and lead to reciprocal chemical shift perturbations upon mutation; their isoleucine cluster (IC) was assigned to three peaks. The resonances of the I59, I89, I235 and I853 δ1-methyl groups are broadened. **b**, Distribution of isoleucine residues in the Xrn2 protein. Ile-δ1 probes are represented as spheres, where assigned probes are colored yellow, and unassigned probes are colored purple. Residues I59, I89, I235, I850 and I853 showed relaxation dispersion and are colored blue; their position is explicitly indicated. I59 and I89 are in close proximity and located above the central β-sheet opposite of I235. I850 and I853 are located in the C-terminal helix of Xrn2, with I853 directly opposite of Y14 at the rear side of the α1-helix. Methyl groups that could only be assigned at 293 K are shown in pink. **c**, MQ CPMG RD profiles measured at 313 K and 500 (yellow), 600 (red) and 800 (purple) MHz ^1^H frequency. Data points are shown with error bars derived from multiple measurements; the curve corresponds to the best fit of the joined analysis of MQ CPMG and ^13^C-SQ CPMG data from all five residues. Fit values for | Δω_C_ | are given in the individual panels. Data points are shown as mean ± s.d., as derived from at least two duplicate NMR measurements.

**Extended Data Fig. 3:**
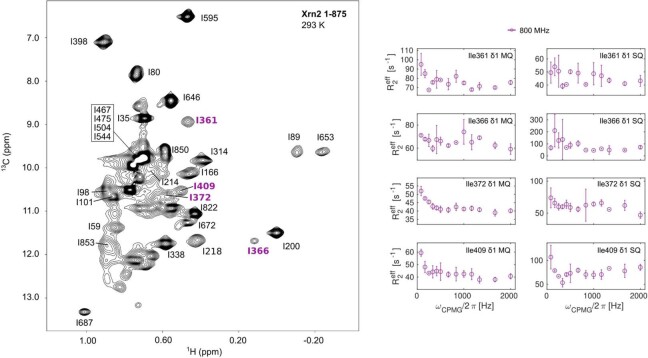
^1^H-^13^C methyl TROSY spectrum of Xrn2 at 293 K and exemplary CPMG RD curves. Methyl TROSY NMR spectrum of Xrn2 recorded at 293 K. Resonances that could only be assigned at 293 K are indicated in magenta. At 313 K these resonances could not be assigned due to strong exchange broadening. The residues that appear at 293 K display elevated relaxation rates at lower CPMG frequencies in both ^13^C SQ and ^1^H-^13^C MQ CPMG RD experiments. Due to overall enhanced relaxation and increased peak overlap at 293 K, a faithful fit of the CPMG RD data could not be reliably performed at the lower temperature. Data points are shown as mean ± s.d., as derived from 2 duplicate NMR measurements.

**Extended Data Fig. 4:**
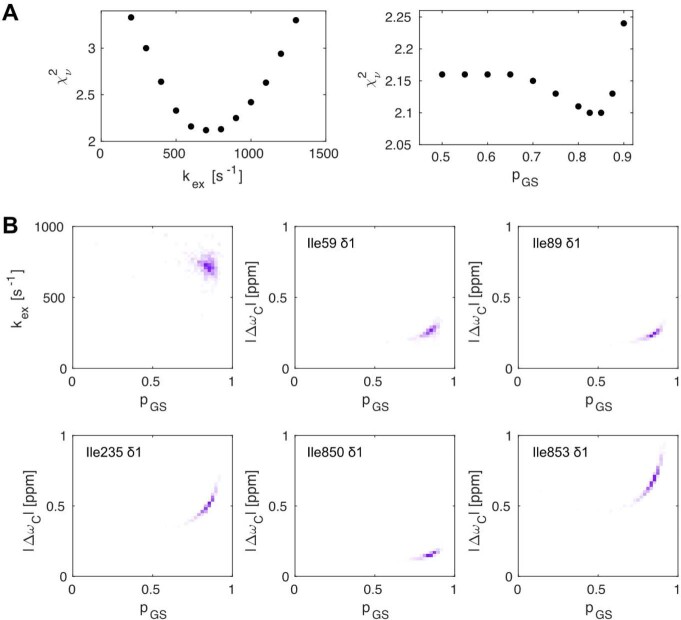
Reduced χ^2^ surfaces obtained from constrained parameter optimization and distribution of fit parameters obtained from Monte Carlo trials: ^13^C data. (A) One dimensional reduced χ^2^ surfaces for ^13^C (SQ and MQ) CPMG RD data, where the fit is restricted by the exchange rate k_ex_ (left) or the ground state population p_GS_ (right). The reduced χ^2^ as a function of k_ex_ exhibits a well defined minimum around 700-800 s^−1^, indicating that the exchange rate is well defined by the experimental data. The plot of reduced χ^2^ as a function of p_GS_ on the other hand reveals a shallow minimum around p_GS_ = 82%. In the current exchange regime the precision with which p_ES_ can be determined strongly depends on the lowest CPMG frequency that can be recorded, which is in turn limited by the fast relaxation rates of the very large enzyme. Note that p_GS_ is well defined towards higher ground state populations (that is χ^2^ strongly increases for larger p_GS_), but loosely defined towards lower ground state populations (that is χ^2^ only moderately increases for lower p_GS_). (B) Subplots show the distribution of the exchange constant k_ex_ versus the ground state population p_GS_, as well as versus the carbon chemical shift differences | ΔωC | for the isoleucine residues that were used in the fitting. The distributions are based on a global fit of two RD experiments (SQ and MQ) for the 5 indicated residues at 3 magnetic field strengths (500, 600 and 800 MHz proton frequency). Note the typical correlation between Δω and pGS in the residues specific plots. The extracted fitting parameters, including errors are summarized in Supplementary Table 6.

To elucidate these and other structural dynamics in Xrn2, we turned to a methyl-TROSY (transverse relaxation-optimized spectroscopy) NMR approach. Heteronuclear multiple quantum coherence (HMQC) spectra of Ileδ_1_-[^13^CH_3_]-labeled and Metε-[^13^CH_3_]-labeled Xrn2 samples are of very high quality, especially when considering that Xrn2 is a 100-kDa single-chain protein (Fig. 2a). To be able to reveal site-specific information, we assigned M704 as well as 38 of the 48 isoleucine resonances (Supplementary Table 2). A subset of these isoleucine probes showed substantial peak broadening at 313 K and could only be assigned at 293 K (Fig. 2b and Extended Data Fig. 3). Importantly, we noted that the resonances that broaden at higher temperature cluster around the α1-helix, which indicates that this region of the enzyme undergoes a conformational exchange process on the microsecond to millisecond time scale. To verify this hypothesis, we recorded methyl group single-quantum (SQ) and multiple-quantum (MQ) Carr–Purcell–Meiboom–Gill (CPMG) relaxation dispersion (RD) experiments at proton Larmor frequencies of 500 MHz, 600 MHz and 800 MHz (Supplementary Fig. 9). These experiments can be used to quantify motions for which the lifetime of the excited state is on the order of milliseconds. We found strong relaxation dispersions in several resonances that surround the α1-helix and could fit all six datasets from five probes simultaneously with the same exchange parameters p_ES_ = 18.1 ± 6.2% and k_ex_ = 710 ± 63 s^−1^ (where p_ES_ is the population of the excited state, and k_ex_ is the exchange rate) (Fig. 2c, Supplementary Table 6 and Extended Data Fig. 4). The precision of the extracted population is limited, as can be judged from the shallow minimum in the reduced χ^2^ surface (Extended Data Fig. 4). Taken together, our data establish that the α1-‘ratchet’-helix of Xrn2 samples multiple states in solution in the absence of a substrate, whereas other parts of the enzyme are largely devoid of motions on the millisecond time scale (Supplementary Fig. 10)

### ^19^F NMR provides an independent measure of α1-helix dynamics

**Fig. 3:**
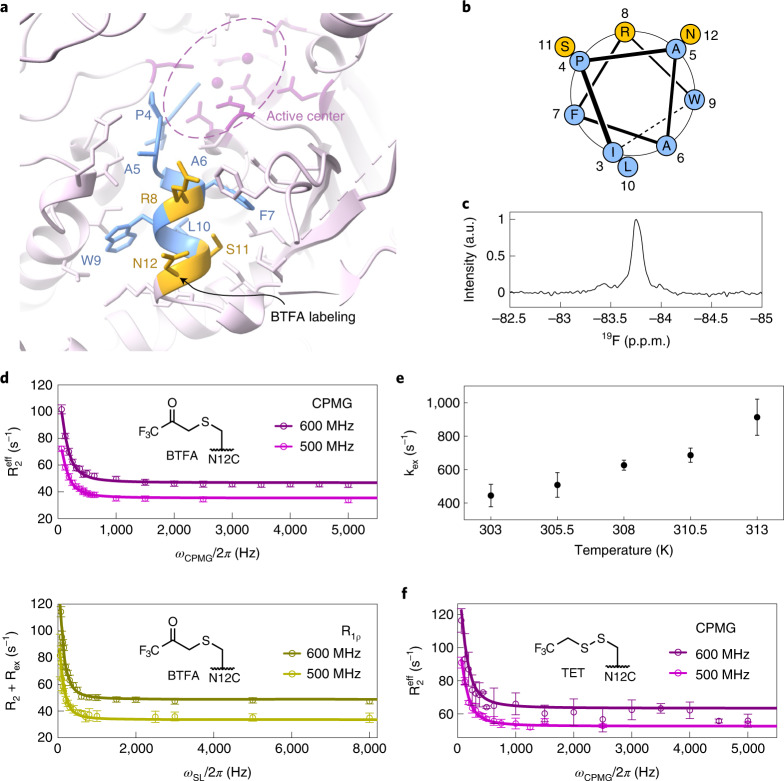
^19^F NMR supports the sampling of an excited conformational state by the α1-helix. **a**, Close-up view of the α1-helix, where the mutation N12C was introduced to allow for BTFA labeling. Hydrophobic residues are shown in blue; charged and polar solvent-exposed residues are shown in yellow. **b**, Helical wheel projection for residues 3–12 in the α1-helix. Coloring as in **a**. **c**, ^19^F NMR spectrum of Xrn2 ΔZnF N12C^BTFA^. **d**, CPMG and on-resonance R_1ρ_ dispersion profiles for Xrn2 ΔZnF N12C^BTFA^ samples recorded at 500 MHz and 600 MHz ^1^H frequency at 313 K. Data points are shown with error bars derived from multiple measurements; lines show the best fit derived from the simultaneous analysis of all datasets from five temperatures with one global | Δω | . At 313 K, this yielded: p_GS_ = 0.50 ± 0.06; k_ex_ = 913 ± 108 s^−1^; and | Δω | = 0.15 ± 0.01 p.p.m. (Supplementary Table 6). Data points are shown as mean ± s.d., derived from three duplicate NMR measurements. **e**, Correlation between the exchange rate (k_ex_) and the temperature. Note that the recording of the ^19^F relaxation data is considerably faster than the recording of the ^13^C data, as the latter depends on a series of 2D NMR spectra. Data points are shown as mean ± s.d., derived from 500 Monte Carlo simulations (Extended Data Fig. 5, Supplementary Figs. 12 and 13 and Supplementary Table 6). **f**, CPMG dispersion profiles of Xrn2 ΔZnF N12C^TET^ at 11.7 T (500 MHz ^1^H frequency) and 14.1 T (600 MHz ^1^H frequency) at 313 K. The data were fit with the population p_GS_ = 50% and the exchange constant k_ex_ = 913 s^−1^ obtained from analysis of the BTFA data. Data points are shown as mean ± s.d., derived from three duplicate NMR measurements. a.u., arbitrary unit.

The methyl CPMG RD experiments provided accurate information with a high spatial resolution. However, one drawback of those experiments is the extended measurement time that is required (>2 days per dataset). This prevents the execution of NMR measurements in the presence of substrates, as these would be turned over before the acquisition of the data is finished. Recently, we showed that ^19^F RD data can be recorded in substantially less time and that this approach can provide accurate insights into bio-molecular exchange processes^38^. Here, we introduced a cysteine in Xrn2 at position 12 that is located at the end of the α1-helix and exposed on the surface (Fig. 3a,b), and we labeled this residue with bromotrifluoroacetone (BTFA) (Supplementary Fig. 11).

**Extended Data Fig. 5:**
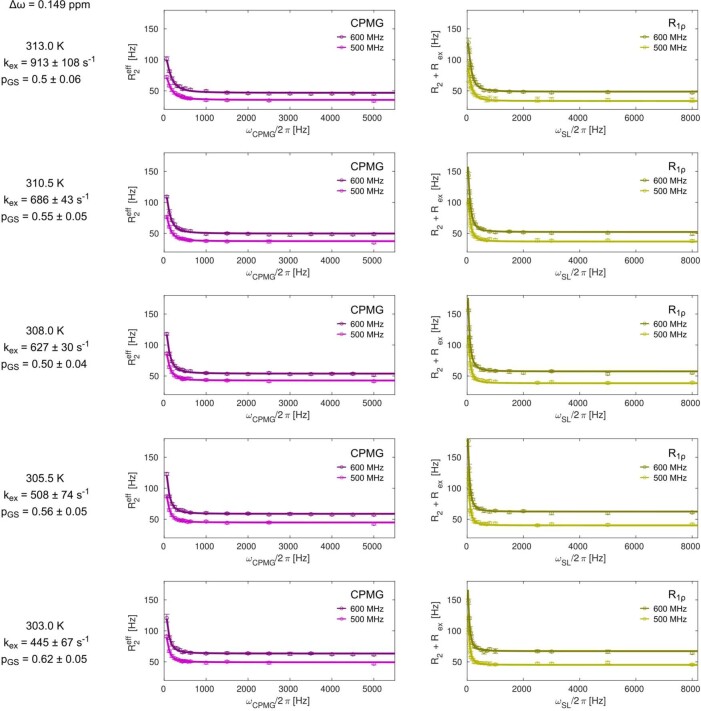
Temperature dependent ^19^F relaxation dispersion. ^19^F CPMG (left) and R_1ρ_ (right) relaxation dispersion of CtXrn2 1-875 ΔZnF N12C^BTFA^ at temperatures between 303 K and 313 K and ^1^H Larmor frequencies of 500 MHz and 600 MHz. Fit parameters of the global fit and separate fits of CPMG and R_1ρ_ datasets are included in Table S6. Note that the R_1ρ_ data contains more datapoints at low frequencies. In the CPMG experiments the number of possible frequencies is determined by the relaxation delay and can only be a multiple of 1 / T_relax_ = 62.5 Hz (for the T_relax_ of 16 ms that was used). The R_1ρ_ experiment, on the other hand, can use arbitrary spin-lock frequencies and is thus able to sample the RD curve at low frequencies better. Note that very low spinlock frequencies cannot be used in R_1ρ_ experiment, as then the signal decay is no longer mono-exponential. The extracted fitting parameters, including errors are summarized in Supplementary Table 6. Data points are shown as mean ± s.d., as derived from 3 duplicate NMR measurements.

**Extended Data Fig. 6:**
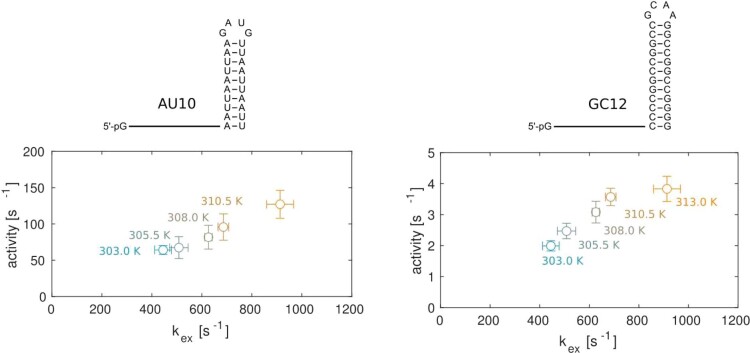
The exchange rate k_ex_ is linearly correlated with the activity in the Xrn2. The activity (turnover rate) and exchange rates (k_ex_) were measured with an Xrn2 ΔZnF N12C-BTFA sample. Activities are derived from HPLC assays, exchange rates are derived from the global fit of 19F relaxation dispersion data (Fig. 3E). The linear correlation between k_ex_ (k_ex_ = k_GS-ES_ + k_ES-GS_) and turnover rates suggests that the exchange and degradation rates are functionally linked. In the apo-state of the enzyme, the population of the excited (active) state is between 20 and 50%. The forward rate (k_GS-ES_) is thus between 0.2*k_ex_ and 0.5*k_ex_. For a 20% excited state the k_GS-ES_ rates (as measured) in the apo-enzyme correlate directly with the turnover rate (activity) of the AU10 RNA, indicating that the translocation of the substrate from the pre-translocation state II to the active state is rate limiting during the catalytic cycle. The degradation rates of RNAs with a stable GC stemloop structure (GC12 RNA) is considerably slower, as the rate limiting step in the catalytic cycle moves from the dynamics in the N-terminal helix to the time it takes to melt the secondary structure in the RNA substrate. Data points are shown as mean ± s.d. The errors in the activities are derived from 2 to 4 independent experiments; the errors in the rates are derived from 500 MC simulations (Extended Data Fig. 5, Supplementary Figs. 12 and 13 and Table S6). Source data

**Extended Data Fig. 7:**
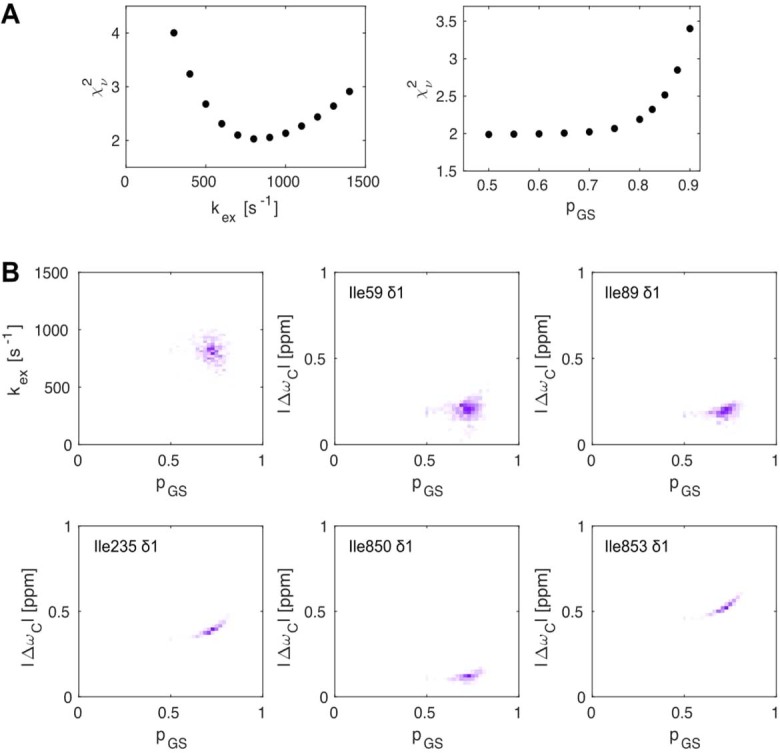
Reduced χ^2^ surfaces obtained from constrained parameter optimization and distribution of fit parameters obtained from Monte Carlo trials: simulations for ^19^F and ^13^C RD data. (A) One dimensional reduced χ^2^ surfaces for the simultaneous analysis of ^13^C CPMG, ^19^F CPMG and ^19^F R_1ρ_ relaxation data. Reduced χ^2^ values were obtained as a function of the exchange rate and the ground state population after optimization of all remaining free parameters. (B) Distribution of fit parameters obtained from MC simulations, where ^13^C CPMG, ^19^F CPMG and ^19^F R_1ρ_ relaxation data were fitted simultaneously. The extracted fitting parameters, including errors are summarized in Supplementary Table 6.

Based on time-efficient one-dimensional (1D) ^19^F NMR spectra (Fig. 3c), we recorded CPMG and on-resonance R_1ρ_ relaxation dispersion data at ^1^H Larmor frequencies of 500 MHz and 600 MHz and at five temperatures between 303 K and 313 K (Fig. 3d,e and Extended Data Fig. 5). For all temperatures, we observed strongly enhanced effective transverse relaxation rates at CPMG/spin-lock frequencies below 500 Hz. We fit all data simultaneously with a single global chemical shift difference parameter | Δω | and temperature-dependent parameters for excited state populations p_ES_^temp^ and exchange rates k_ex_^temp^ (Supplementary Figs. 12 and 13). We obtained | Δω | = 0.15 ± 0.01 p.p.m. and found that k_ex_ increased from 445 ± 67 s^−1^ to 913 ± 108 s^−1^ between 303 K and 313 K (Fig. 3e and Extended Data Fig. 6). The population of the excited state is high (> ~20%) for all temperatures, although it is not possible to determine p_ES_ with high accuracy, as was the case for the ^13^C data. We note that the CPMG data contain less information than the on-resonance R_1ρ_ data, as, for the latter, it is possible to record more data points at low (spin-lock) frequencies that are most sensitive to the exchange parameters (Supplementary Fig. 12 and Supplementary Table 6). The finding that the motions in Xrn2 are localized to a region around the active site is corroborated by measurements on samples where the ^19^F label has been positioned at other sites in Xrn2 (Supplementary Fig. 14). In addition, we found that ^19^F RD profiles that we recorded for a specific position are the same for enzymes that have been labeled using BTFA or trifluoroethanethiol (TET) (Fig. 3f). Together, these observations establish that our ^19^F RD profiles are suitable for detecting dynamic processes of the region around the active site of Xrn2. The exchange rates at 313 K (k_ex_) that we extract from the ^19^F data and from the methyl-TROSY data at 313 K are compatible (k_ex_^CH3^ = 710 ± 63 s^−1^ and k_ex_^19F^ = 913 ± 108 s^−1^), whereas p_ES_ is, in both cases, relatively high. The consistency of the ^13^C and ^19^F measurements is further confirmed by a global fit of the ^13^C and ^19^F RD data (Extended Data Fig. 7), from which we obtained an exchange rate of 864 ± 112 s^−1^ (Supplementary Table 6).

### Substrate binding changes the structure and dynamics of Xrn2

The fast degradation of RNA by Xrn2 prevents the NMR characterization of a complex between Xrn2 and an RNA substrate. To investigate the structural changes that occur in Xrn2 upon substrate interaction, we used three substrates with strongly attenuated degradation rates: 3′,5′-bisphosphoadenosine (pAp), a 5-mer DNA and an exoribonuclease-resistant RNA (xrRNA)^39^. It is important to note that we use the catalytically active WT Xrn2, as versions of the enzyme that lack the catalytically important magnesium ions likely display altered interactions and/ or motions.

**Fig. 4:**
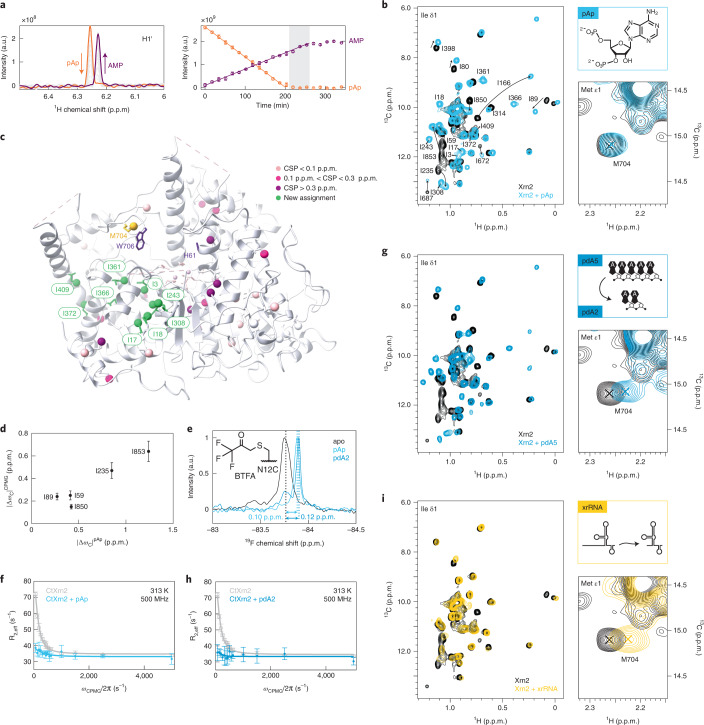
Substrate binding induces a conformational change to a more rigid Xrn2 state. **a**, Left: ^1^H NMR spectrum showing the H1' resonance of pAp (orange) and AMP (purple). Right: linear fit of the integrated peak intensities reveals a turnover rate of 0.05 min^−1^. Data points are shown as integrated peak intensities; error bars represent spectral noise. **b**, Overlay of methyl-TROSY spectra in the absence (black) and presence (light blue) of pAp. Binding of pAp leads to strong CSPs; the M704 resonance remains almost unperturbed. **c**, Ile-δ1 methyl groups in Xrn2 that could only be assigned in the pAp-bound state are colored green. Ile-δ1 resonances were colored according to the amplitude of the CSP. H61, W706 and M704 are shown as sticks. **d**, Correlation of ^13^C CSPs obtained upon binding of pAp and | Δω_13C_ | extracted from the CPMG data (Fig. 2c). Data points are shown as mean ± s.d., derived from 500 Monte Carlo simulations (Extended Data Fig. 7 and Supplementary Table 6). **e**, ^19^F NMR spectra of Xrn2 ΔZnF N12C^BTFA^ without ligand (black) and bound to pAp (light blue) or pdA5 (dark blue). Upon interaction with the substrates, the ^19^F line width is reduced from 55 Hz to 36 Hz (pAp) and 32 Hz (pdA5), respectively. **f**, CPMG RD profiles of Xrn2 ΔZnF N12C^BTFA^ in the absence (gray) and presence (light blue) of pAp. Data points are shown as mean ± s.d., derived from three duplicate NMR measurements. **g**, Overlay of methyl-TROSY spectra in the absence (black) and presence (dark blue) of pdA5 (that is degraded to pdA2). CSPs are observed in the Ile region; M704 experiences a characteristic shift in the ^1^H dimension. **h**, CPMG RD profiles of Xrn2 ΔZnF N12C^BTFA^ in the absence (gray) and presence (dark blue) of pdA5/pdA2. Data points are shown as mean ± s.d., derived from three duplicate NMR measurements. **i**, Overlay of methyl-TROSY spectra in the absence (black) and presence (yellow) of an xrRNA. The shift of M704 as well as Ile-δ1 CSPs close to the active site and the RNA entry site show that the complex is locked in the pre-translocation conformation. a.u., arbitrary unit.

First, we used pAp^40^, which is hydrolyzed to adenosine 5′-monophosphate (AMP) at a rate of 0.05 min^−1^ (Fig. 4a). This allowed us the measurement of HMQC spectra of Ileδ_1_-[^13^CH_3_]-labeled Xrn2 in the presence of an excess of pAp (Fig. 4b). The binding of pAp to Xrn2 resulted in substantial chemical shift perturbations (CSPs) of the methyl group resonances that are in agreement with a conformational change around the active site of the enzyme (Fig. 4c). Interestingly, the carbon chemical shifts of the excited apo state that we extract from the CPMG experiments (Fig. 2c) correlate linearly with the carbon chemical shifts that we measure in the presence of pAp (Fig. 4d). Likewise, the ^19^F chemical shift difference upon the addition of pAp to Xrn2-deltaZnF-N12C^BTFA^ (0.10 p.p.m.) is in agreement with p_GS_ ∙ |Δω | (= 0.07–0.12 p.p.m. for 0.5 < p_GS_ < 0.8 and | Δω | = 0.149 p.p.m.) that we determined from the RD experiments in the absence of the substrate (Fig. 4e). These findings support the notion that the apo enzyme transiently populates a state that is structurally similar to the pAp-bound conformation. Interestingly, ^19^F line widths and RD experiments reveal that the dynamics in Xrn2 are substantially reduced upon substrate binding (Fig. 4f). Taken together, our data reveal that Xrn2 locks into a stable active conformation (the post-translocation state) upon substrate recruitment. As a consequence of the more rigid nature of the Xrn2:substrate complex, we were able to assign nine additional Xrn2 methyl resonances (Fig. 4c) that were broadened beyond detection in the apo state due to conformational exchange.

To obtain additional information on the structural and conformational changes in Xrn2 upon substrate binding, we next made use of a 5′-phosphorylated DNA pentanucleotide (pdA5) that is readily degraded into a 2-mer DNA (pdA2) and free nucleotides (Supplementary Fig. 15). pdA2 is one nucleotide longer than the pAp that we used above (Fig. 4b) yet induced similar chemical shift perturbations in the HMQC spectrum (Fig. 4g and Supplementary Fig. 16). In analogy to pAp binding, the binding of pdA2 to Xrn2 also abolished the extensive motions in the enzyme (Fig. 4h). pAp and pdA2, thus, both induce the post-translocation conformation in Xrn2. Interestingly, in the presence of pdA2, we noticed a CSP of Met704 that was absent in the pAp-bound states (Fig. 4g). As pdA2 is one base longer than pAp, we concluded that the CSP of Met704 reports on the location of the second base of the Xrn2 substrate. In the structure of Xrn2, Met704 is positioned in direct proximity to the conserved Trp706 residue, which stacks with the third nucleotide base in the structure of Xrn1 bound to a 5′-phosphorylated DNA 11-mer^27,30^. Based on that, Met704 is, thus, an excellent probe that reports on the occupancy of the RNA-binding pocket next to the active site.

**Extended Data Fig. 8:**
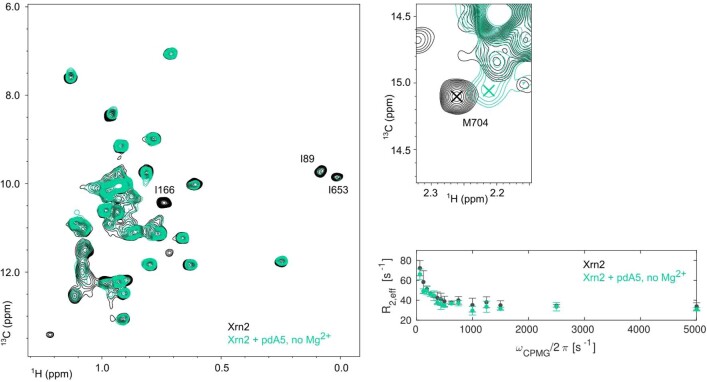
In the absence of Mg^2+^ ions pdA5 interacts with the RNA binding pocket, but does not induce the active conformation in Xrn2. Overlay of the ^1^H-^13^C HMQC methyl-TROSY spectra of the apo state (black) and after addition of pdA5 (green), recorded at 18.8 T and 313 K. CSPs of M704 (right panel) clearly report on the interaction of pdA5 with the RNA-binding pocket. Spectra are recorded in the absence of Mg^2+^. The absence of CSPs around the active site (as observed in the presence of Mg^2+^; Fig. 4G) indicates that the stable active conformation is not formed when Mg^2+^ is not present. Binding of pdA5 in the absence of Mg^2+^ does not change the dynamics of the α_1_-helix (opposed to what was observed in the presence of Mg^2+^; Fig. 4H), as shown by the overlay of ^19^F CPMG data in the absence (black) and presence (green) of pdA5. Data points are shown as mean ± s.d., as derived from 3 duplicate NMR measurements.

In the absence of Mg^2+^, pdA5 is not degraded to pdA2 by Xrn2 (Supplementary Fig. 15). Under these conditions, pdA5 is recruited to the RNA-binding pocket (as reported by Met704; Extended Data Fig. 8), but the active post-translocation conformation in Xrn2 (as reported by the methyl-TROSY spectra; Extended Data Fig. 8) is not stably formed. This is also confirmed by ^19^F RD measurements that show that the binding of pdA5 to Xrn2 in the absence of magnesium has no effect on the dynamics of the N-terminal α1-helix. From that, we conclude that the divalent ions in the active site play an important role in the transition of Xrn2 from the pre-translocation state to the post-translocation state. This finding underscores the importance of performing experiments on the holoenzyme as the Mg^2+^ ions are required for both catalysis and the stable formation of the active post-translocation conformation.

Finally, we used a modified xrRNA derived from a subgenomic flavivirus RNA from Zika virus as an Xrn2 substrate. This RNA contains an unstructured 5′ end followed by a highly stable pseudoknot that is resistant to the 5′→3′ exoribonuclease activity of Xrn1 (ref. ^39^). The addition of Xrn2 to this xrRNA results in the rapid hydrolysis of the unstructured 5′ region and the subsequent formation of a stable complex between Xrn2 and the xrRNA pseudoknot that is sterically hindered to translocate into the active state. (Supplementary Fig. 17). The HMQC spectrum of this complex shows overall reduced peak intensities that are particularly pronounced for resonances of residues between the α3-helix and the active site (Fig. 4I and Supplementary Fig. 18). The reduced resonance intensities can be attributed to the increase in molecular weight and the large number of protons from the xrRNA that come close to Xrn2 methyl groups and, thereby, enhance relaxation rates. Nevertheless, we can conclude that the active post-translocation state of the enzyme is not stably formed, as the signature chemical shifts of the active state (Fig. 4b) are not fully observed. Met704, on the other hand, shows a clear CSP, which confirms that the 5′ end of the trimmed xrRNA interacts in the substrate-binding pocket. Taken together, these data show that the Xrn2 enzyme is locked in a pre-translocation state in the presence of the xrRNA product, as was observed in the cryo-EM structure of Xrn1 in the presence of a stalled ribosome^30^.

In summary, our data reveal that the apo Xrn2 enzyme is in a dynamic equilibrium between the pre-translocation and post-translocation states. Upon recruitment of a substrate and in the presence of Mg^2+^, Xrn2 mainly adopts the post-translocation state.

### The A5F mutation alters Xrn2 dynamics and activity

**Fig. 5:**
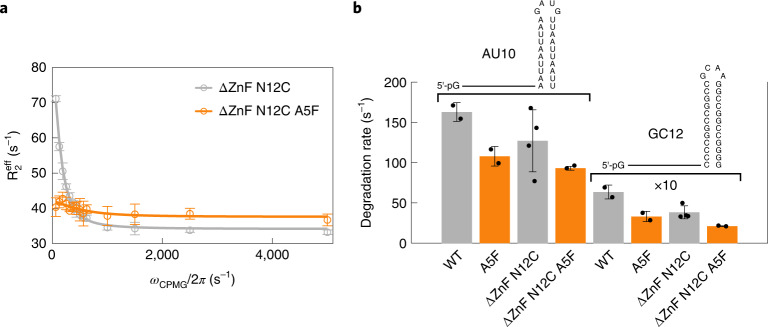
The N-terminal helix is functionally important for RNA degradation. **a**, CPMG relaxation dispersion profiles of Xrn2 ΔZnF N12C^BTFA^ (gray) and Xrn2 ΔZnF A5F N12C^BTFA^ (orange) at 11.7 T (500-MHz ^1^H frequency). Data points are shown as mean ± s.d., derived from three duplicate NMR measurements. **b**, Changes in the degradation rate of 5′-monophosphorylated AU10 and GC12 stem–loop RNAs by Xrn2, the Xrn2 A5F mutant, Xrn2 ΔZnF N12C^BTFA^ and Xrn2 ΔZnF N12C^BTFA^ A5F. The GC12 RNA is degraded approximately 20× slower than AU10, and the degradation rates are multiplied by 10 for clarity. Data points are shown as mean ± s.d., derived from two or four (Xrn2 ΔZnF N12C) independent experiments. Source data

**Extended Data Fig. 9:**
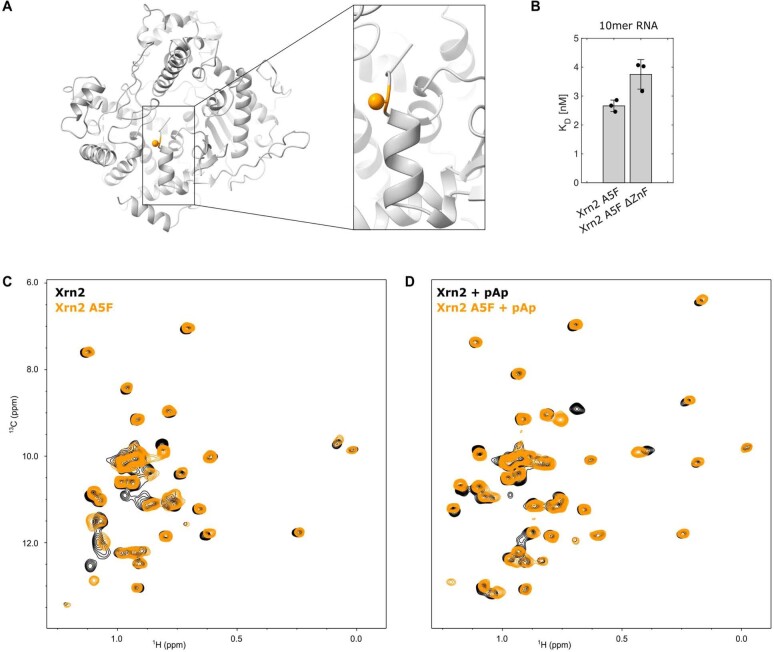
The A5F mutation does not interfere with the Xrn2 structure or with the binding of the substrate to Xrn2. (A) Structure of Xrn2 with the mutation site A5 highlighted in orange and the Cβ atom depicted as a sphere. (B) Xrn2 A5F interacts with a 10mer RNA with very similar affinities as the WT enzyme (compare: Fig. 1G of the main text). Data points are shown as mean ± s.d., as derived from 3 independent experiments. (C) HMQC spectra of the Xrn2 WT (black) and Xrn2 A5F enzyme (orange). (D) HMQC spectra of the Xrn2 WT:pAp complex (black) and Xrn2 A5F:pAp complex (orange). Minor CSPs are visible around the N-terminal α1-helix, that are due to the A5F point mutation. The overall structural of the active state is, however, maintained in the presence of the A5F mutation (the black and orange spectra are highly similar in the absence and presence of substrate). Source data

To investigate how the dynamics in Xrn2 are correlated with RNA substrate degradation, we introduced single point mutations in or close to the α1-helix with the aim of changing the dynamics around the active site (Supplementary Table 5). We found that the A5F mutation led to substantially reduced relaxation dispersions (Fig. 5a), indicative of substantial changes in the energy landscape of the enzyme. It is important to note that this mutation does not interfere with the overall structure of the enzyme or with the interaction between the substrate and the enzyme (Extended Data Fig. 9). These data can be explained by a higher energy barrier between the pre-translocation and post-translocation states. Interestingly, we found that the A5F mutation decreases the activity of Xrn2 by 30–50%, depending on the substrate (Fig. 5b). Based on these findings, we conclude that the A5F mutation results in changes in the dynamics of Xrn2 and, at the same time, in a decrease in the enzymatic turnover rates.

### Regulation of Xrn2 activity

**Extended Data Fig. 10:**
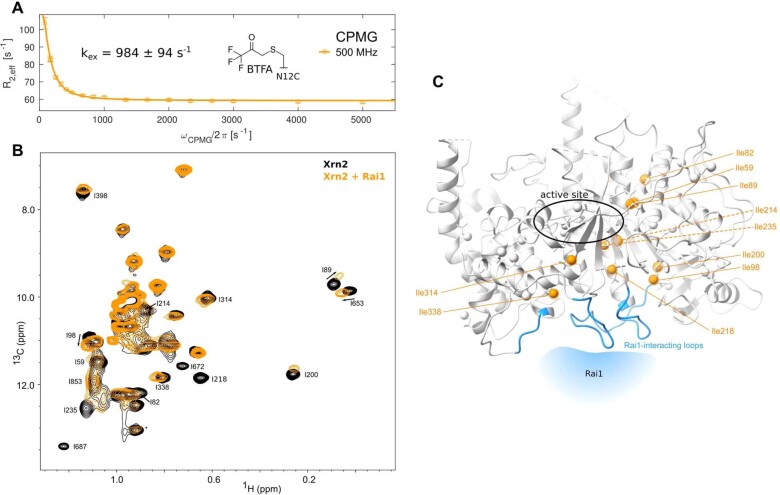
Changes in the Xrn2 enzyme spread from the Rai1 interface along the central β-sheet. (A) ^19^F CPMG RD profile of Xrn2 ΔZnF N12C^BTFA^:Rai1 sample at 313 K. The fit yields an exchange rate of k_ex_ = 984 ± 94 s^−1^, which matches the exchange rate of Xrn2 ΔZnF N12C^BTFA^ in the absence of Rai1 (k_ex_ = 913 ± 108 s^−1^). Binding of Rai1 thus does not influence the motions in Xrn2. Data points are shown as mean ± s.d., as derived from 3 duplicate NMR measurements. (B) HMQC spectrum of Ileδ_1_-[^13^CH_3_] and Metε-[^13^CH_3_] methyl labeled Xrn2 in the absence (black) and presence (orange) of unlabeled, protonated Rai1. The resonances in the active site of Xrn2 are not influenced, indicating that Rai1 does not affect the active site of Xrn2. (C) Structure of Xrn2. Ileδ_1_ methyl groups that show pronounced changes in the HMQC spectrum upon interaction with Rai1 are highlighted in orange. The putative Rai1 interaction surface, inferred from the structure of the Xrn2:Rai1 complex in *S. pombe* (PDB 3FQD), is highlighted in blue. CSPs upon Rai1 interaction reach from the Rai1 binding site towards a region that is remote from the active site. pronounced changes in the HMQC spectrum upon interaction with Rai1 are highlighted in orange. The putative Rai1 interaction surface, inferred from the structure of the Xrn2:Rai1 complex in *S. pombe* (PDB 3FQD), is highlighted in blue. CSPs upon Rai1 interaction reach from the Rai1 binding site towards a region that is remote from the active site.

In previous studies, it was shown that the activity of Xrn2 can be enhanced by the interaction with binding partners^25,41^. On the one hand, Xrn2 in higher eukaryotes can recruit XRN2-binding domain (XTBD) containing binding partners that have been proposed to stabilize the Xrn2 fold in the absence of a substrate^37^. However, in our crystal structure of apo CtXrn2, this XTBD segment is already fully folded (Supplementary Fig. 19), and the chemical shifts of Ile687 and Ile688 δ1-methyl groups clearly deviate from the shift of Ileδ1-methyl groups in unstructured regions (Fig. 2a). As XTBD-containing proteins have only been identified in metazoa^37,41^, our findings suggest that the mechanisms that regulate the catalytic activity of Xrn2 differ between yeast and metazoa. On the other hand, yeast Xrn2 directly interacts with the pyrophosphohydrolase Rai1 in *S. pombe;* this interaction enhances the activity of the enzyme due to the Rai1-induced stabilization of the Xrn2 fold^25^. Xrn2 and Rai1 also form a stable complex in *C. thermophilum* (Supplementary Fig. 20); however, we observe that the recruitment of CtRai1 to CtXrn2 does not result in an enhancement of Xrn2 activity (Supplementary Fig. 21), and neither are the structure or motions in the active site of Xrn2 influenced by Rai1 binding (Extended Data Fig. 10). In *S. pombe*, Rai1 enhances Xrn2 activity especially for RNA substrates that contain stable secondary structure elements, indicating that Rai1 possesses RNA unwinding activity. In *C. thermophilum*, this unwinding activity appears to have moved from the Xrn2-interacting protein Rai1 to the ZnF in the enzyme.

## Discussion

Static macromolecular structures provide important information about biochemical mechanisms. It is, however, important to realize that these static structures (1) do not necessarily represent the ground state conformation of the macromolecule in solution^42^ and (2) fail to reveal the dynamical processes that underlie allostery^43,44^ or catalysis^45–47^. In that regard, it is, thus, of central importance to complement our understanding of molecular function with information regarding dynamic processes, although this can be technically highly challenging^48^.

A catalytic cycle of a chemical reaction comprises multiple steps, where the overall rate of the reaction is limited by the slowest of these steps. Enzymes can increase the speed of these reactions substantially, by reducing the rate-limiting step that typically involves the breaking or formation of chemical bonds. In an enzyme-catalyzed chemical reaction, the slowest step can then change from the chemical step to a conformational change in the enzyme. This has been shown to be the case for, for example, dihydrofolate reductase (DHFR), which needs to rearrange from a closed conformation where catalysis can take place to an occluded conformation that is required for product release^49^. Likewise, the opening of a lid domain was found to constitute the rate-limiting step in the reaction cycle of hyperthermophilic and mesophilic homologs of the enzyme adenylate kinase^50^.

In this study, we exploited methyl-TROSY (Figs. 2 and 4) and ^19^F (Figs. 3, 4 and 5) NMR techniques, and we show that the N-terminal α1-helix in the 5′→3′ exoribonuclease Xrn2 is highly dynamic. In the apo state, the enzyme adopts the pre-translocation (ground) state (Fig. 1a and Supplementary Fig. 2) between 50% and 80% and the post-translocation (active/excited) state between 20% and 50% (k_ex_ = 864 s^−1^; 173 s^−1^ < k_GS-ES_ < 432 s^−1^ and 432 < k_ES-GS_ < 691 s^−1^).

Substrate binding shifts the conformational equilibrium to the post-translocation state, likely by reducing the free energy of this conformation. As a result, the backward rate (k_ES-GS_) will be substantially reduced (to less than 10 s^−1^) while the forward rate (k_GS-ES_) rate remains unchanged. This scenario is in full agreement with the changes in the ^19^F RD profile that we observe upon substrate binding (Fig. 4f,h). Interestingly, for RNA substrates without strong secondary structure elements (AU hairpin RNA), the turnover rate (127–163 s^−1^) is close to the rate with which the enzyme transitions from the pre-translocation state to the post-translocation state (k_GS-ES_). This suggests that this transition can constitute the rate-limiting step in the catalytic cycle. This notion is further corroborated by the observation that k_cat_ and k_ex_ both linearly change with temperature (Extended Data Fig. 6). Finally, the central importance of the dynamics of the N-terminal helix is revealed by the A5F mutant enzyme that results in changes in the energy landscape of Xrn2 (Fig. 5) that coincide with a reduction in the turnover rates.

In summary, we made extensive use of NMR methods to quantify and localize structural changes in Xrn2. It should be noted that Xrn2 (875 residues, 100 kDa) is among the largest single-chain enzymes that have so far been studied using solution-state NMR methods. We exploited methyl labeling strategies, which provide a higher spatial resolution, and ^19^F labeling, which is able to provide information regarding protein motions in short times. It is important to note that positioning of the fluorine label was directly guided by data obtained from the methyl-labeled samples. We found that an approach that combines methyl group and fluorine labeling is highly efficient. However, we would also like to stress the importance of cross-checking that the methyl and fluorine labels report on the same molecular process. Here, we ensured this by comparing the exchange parameters that were extracted from ^13^CH_3_-based and ^19^F-based RD measurements and by labeling the enzyme with two independent fluorine-containing compounds (BTFA and TET). Importantly, the rapid (1-hour) ^19^F experiments allowed us to obtain information on catalytically active Xrn2 in the presence of substrates, before those were fully processed. We anticipate that the combination of complementary NMR labeling techniques together with information on static structures will be an important approach to obtain fundamental insights into biologically important dynamics in large enzymes.

## Methods

### Molecular biology

The protein-coding sequence of Xrn2 from *C. thermophilum* (National Center of Biotechnology Information (NCBI) reference sequence XP_006691410.1) was synthesized by GenScript with codon optimization for expression in *Escherichia coli* and provided in a pUC57 vector. A construct encompassing amino acids 1–875 (Supplementary Table 1) was cloned into a pET26b^+^ vector (Novagen) using NdeI and XhoI restriction sites (hereafter referred to as CtXrn2). The vector carries no additional amino acids at the N-terminus of the construct and a C-terminal His_6_-tag constituted by the sequence LEHHHHHH, where the amino acids LE are a remnant from the restriction site.

A construct lacking the residues 263–300, corresponding to the ZnF loop (hereafter referred to as CtXrn2 ΔZnF) was prepared using Gibson assembly cloning. All point mutations were introduced using site-directed mutagenesis with a pair of primers designed with PrimerX (http://www.bioinformatics.org/primerx), where the mutated sequence was typically introduced in the middle of the primers. A construct containing the ZnF (residues 265–293) was cloned using Gibson assembly into a pETGB1a vector that carried an N-terminal His6-GB1 tag, followed by a tobacco etch virus (TEV) protease cleavage site. All used constructs were validated by sequencing the full open reading frame.

### Protein expression and purification

Plasmids were transformed into *E. coli* BL21(DE3) CodonPlus-RIL cells (StrataGene) and grown in LB overnight at 37 °C. For unlabeled (non-isotope-labeled) samples, typically 1 L of LB was inoculated from an overnight culture and grown to an OD_600_ of 0.6–0.8 at 37 °C, at which point the culture was cooled to 20 °C, and protein expression was induced by the addition of 0.5 mM IPTG. For samples with Ile-δ_1_^13^C^1^H_3_ methyl group labeling, an H_2_O-M9 culture was inoculated from the overnight LB culture and grown to OD ~0.2–1.0. Cells of this culture were harvested typically in the afternoon by moderate centrifugation at ~3,000 relative centrifugal force (r.c.f.) and resuspended in D_2_O-M9 (containing 2 g L^−1^ of ^2^H^12^C-labeled glucose) culture to an OD of 0.1–0.15 with a volume of 1/10 of the final D_2_O culture volume. The D_2_O-M9 culture was grown overnight at 37 °C, diluted 1:8 and grown to an OD of 0.6–0.8. A solution of the isoleucine precursor alpha-ketobutyrate in a buffered D_2_O solution was added to the culture; the temperature was shifted to 20 °C; and protein expression was induced 1 hour later by the addition of 0.5 mM IPTG. Cells were harvested by centrifugation 14–18 hours after induction, and cell pellets were stored at −20 °C. Protein purification followed a two-step protocol with a Ni-affinity chromatography and a gel filtration. In brief, cell pellets were resuspended in 50 mM Na phosphate buffer (pH 7.4), 400 mM NaCl, 10 mM imidazole, 0.1% Triton X-100, 1 mg L^−1^ of lysozyme and 0.2 U ml^−1^ of DNase I with vortexing for 30 minutes at 4 °C and lysed by sonication. The cell debris was removed by a centrifugation step at 18,000 r.c.f., and the supernatant was cleared with a 0.45-μm syringe filter and loaded on a gravity flow Ni-NTA column with a typical volume of 2 ml of beads, which was pre-equilibrated with buffer A (50 mM Na phosphate (pH 7.4), 400 mM NaCl and 10 mM imidazole). The column was subsequently washed with at least 10 column volumes of buffer A, and the bound protein was eluted with typically 10–15 ml of buffer B (50 mM Na phosphate (pH 7.4), 150 mM NaCl and 300 mM imidazole), until a Bradford test did not show staining any more, and 1 mM DTT was added to the elution. The elution was concentrated to a volume of 1–1.5 ml and purified by size-exclusion chromatography using a 16/600 Superdex S200 column in GF buffer (25 mM HEPES (pH 7.3), 125 mM NaCl and 1 mM DTT). The target fractions were combined and concentrated. For deuterated samples, the buffer was exchanged in this concentration step by multiple rounds of addition of D_2_O-GF buffer (25 mM HEPES (pH 7.3), 125 mM NaCl and 1 mM DTT in D_2_O) and subsequent concentration. NMR samples were supplemented with 0.03% NaN_3_ and 5% D_2_O (for samples not in 100% D_2_O).

The ^13^C/^15^N-labeled ZnF was expressed in M9 medium containing 0.5 g L^−1^ of ^15^NH_4_Cl and 2 g L^−1^ of ^1^H^13^C-labeled glucose. The purification followed the procedure described above but included a dialysis step against 50 mM Na phosphate (pH 7.4), 150 mM NaCl and 1 mM DTT with simultaneous TEV protease cleavage after the Ni-affinity chromatography step. The cleaved His_6_-GB1 tag was removed by applying the solution to a second Ni-affinity chromatography step, where the resin was equilibrated in buffer A. The flow-through of the column was collected, concentrated and purified using a 16/600 Superdex S75 column equilibrated in GF buffer. Target fractions were pooled and concentrated again, and the sample was supplemented with 0.03% NaN_3_ and 5% D_2_O.

### Crystal structure determination

Crystallization of *C. thermophilum* Xrn2 1-875 was carried out with vapor diffusion in sitting drops, derived from 0.3 μl of Xrn2 (36 mg ml^−1^) in 25 mM HEPES (pH 7.3), 125 mM NaCl, 0.5 mM DTT and 0.3 μl of the precipitant reservoir solution (20% w/v PEG3350 and 200 mM magnesium formate). Crystals grew as thin needles within 2–4 weeks. Crystals were shortly incubated in the reservoir solution with 30% glycerol added for cryoprotection before harvesting. Diffraction data were collected at 100 K on the PXII beamline at the Swiss Light Source with a wavelength of 1 Å and processed with XDS^51^. *S. pombe* Xrn2 (Rat1, Protein Data Bank (PDB) entry 3FQD) was pruned with the CCP4 utility Chainsaw to serve as a search model for molecular replacement, which was performed with the CCP4 utility Phaser^52^. The final structure was obtained after performing several cycles of iterative model building and refinement with Coot^53^ and Phenix^54^, respectively. Structure factors and coordinates have been deposited in the PDB under accession code 7OPK.

### Cysteine fluorine labeling

Proteins were fluorine labeled after gel filtration at protein concentrations of typically 100 μM. For labeling with 3-bromo-1,1,1-trifluoroacetone (BTFA), the sample was cooled on ice, and BTFA was added to a final concentration of 10 mM. The reaction was incubated for 30 minutes on ice and quenched with 20 mM DTT, and the sample was purified over a PD10 or PD Minitrap G-25 desalting column pre-equilibrated with GF buffer. NMR samples were supplemented with 0.03% NaN_3_ and 5% D_2_O. For labeling with TET, the sample was cooled to 4 °C; dichloro-(1,10-phenanthroline)-copper (II) (CuPh) was added to a final concentration of 20 μM; and TET was added to a final concentration of 500 μM. The reaction was followed in ^19^F spectra until free TET was depleted.

### RNA preparation

5′-monophosphorylated RNAs were prepared by in vitro transcription (IVT) in the presence of an excess of GMP over GTP. In brief, a template DNA oligonucleotide with the desired RNA sequence in reverse complement followed by a reverse complementary T7 promoter sequence at the 3′ end (5′-TATAGTGAGTCGTATTACG-3′; Supplementary Table 4) was used in equimolar amounts with an antisense T7 promoter oligonucleotide (5′-CGTAATACGACTCACTATAGG-3′) in the reaction at a concentration of 1 μM. The reaction included 40 mM Tris (pH 8.0), 5 mM DTT, 1 mM spermidine, 0.01% Triton X-100, 10–40 mM MgCl_2_ and 0.2 μM T7 RNA polymerase (prepared in-house). GMP was included at a concentration of 10 mM, and the required nucleoside triphosphates were included typically at 4 mM concentrations, with the exception of GTP, which was used at a concentration of 0.8 mM. IVTs were carried out at 37 °C for 4 hours to overnight, after which 50 mM EDTA was added to dissolve the magnesium-pyrophosphate precipitate. Reaction products were precipitated with 0.7 reaction volumes of isopropanol and 0.1 reaction volumes of 3 M NaOAc (pH 5.3) with subsequent cooling at −20 °C for at least 30 minutes. The precipitate was pelleted by centrifugation, washed with cold 70% ethanol, air dried and resuspended in 5 M urea and 20 mM Tris (pH 8.0). The solution was applied to a preparative anion exchange DNAPac column (Dionex) and separated by size with an NaCl gradient. Fractions containing RNA were analyzed with urea polyacrylamide gel electrophoresis, and the fractions of interest were pooled, precipitated with isopropanol, washed with 70% ethanol and resuspended in H_2_O. If necessary, the solutions were concentrated with a SpeedVac vacuum concentrator. RNA samples were stored at −20 °C.

For fluorescence anisotropy measurements, RNAs (5-mer 5′-GGAGU-3′ and 10-mer 5′-GGAGGAGAGU-3′) were fluorescently labeled with 6-iodacetamido-fluorescein. To this end, IVTs were carried out in the presence of 200 μM 4-thiouridine, and the RNA was precipitated, washed with 70% ethanol, resuspended in 0.1 M sodium phosphate (pH 8.0) and incubated with 10 mM iodoacetamido-fluorescein at room temperature overnight. The RNA was twice precipitated and resuspended in H_2_O and purified with a PD MiniTrap column pre-equilibrated with 20 mM Tris (pH 8.0). The RNAs were cleaved after the labeled thiouridine residue with RNase A at 37 °C for 30 minutes and purified by urea polyacrylamide gel electrophoresis.

### Activity assays

RNase assays were performed in GF buffer (25 mM HEPES (pH 7.3), 125 mM NaCl and 1 mM DTT) with RNA concentrations of 10–20 μM and protein concentrations of 5–125 nM. Protein samples used for activity assays were prepared freshly and mixed with 0.01% Triton X-100. Next, 10-μl samples were stopped with 30 μl of 8 M urea, after which the samples were immediately heated to 90 °C for 2 minutes and stored at −20 °C for high-performance liquid chromatography (HPLC) analysis. HPLC assays were performed at 40 °C on a DNAPac PA100 column (Dionex) using an HPLC system with automatic injection of 18-μl samples. Substrate and products were separated using a linear NaCl gradient from 5 M urea, 20 mM Tris/HCl (pH 8.0) and 100 mM NaCl to 5 M urea, 20 mM Tris/HCl (pH 8.0) and 600 mM NaCl. For qualitative assays, reactions were stopped using an equivalent volume of 2× RNA loading buffer (8 M urea, 20 mM EDTA (pH 8.0), 2 mM Tris (pH 8.0), 0.025% w/v bromophenol blue and 0.025% w/v xylene cyanole) and analyzed by urea polyacrylamide gel electrophoresis. Activity assays were performed 2–4 times with fully independently purified enzymes. The indicated error bars are the standard deviations that are based on the independent measurements. The raw data can be found in the online Source Data file.

### NMR spectroscopy

HMQC SOFAST spectra were recorded with a Bruker Avance III 800-MHz spectrometer equipped with a helium-cooled TCI probehead, unless indicated otherwise. SQ-CPMG and MQ-CPMG^55^ experiments were recorded with Bruker Avance NEO 500-MHz, 600-MHz and 800-MHz spectrometers equipped with helium-cooled (800 MHz) or nitrogen-cooled (500 MHz and 600 MHz) TCI probeheads as pseudo-3D experiments, where the CPMG frequency was used as a third dimension that was recorded in an interleaved manner. Data were recorded with a recycle delay of 1.5 seconds and carbon acquisition times of 24.8 ms (800 MHz), 29.0 ms (600 MHz) and 29.8 ms (500 MHz). In all CPMG experiments, the constant time T_CPMG_ was set to 24 ms, and frequencies were sampled at 83.3 Hz, 166.7 Hz, 250 Hz, 333.3 Hz, 416.7 Hz, 500 Hz, 666.7 Hz, 833.3 Hz, 1,000 Hz, 1,166.7 Hz, 1,333.3 Hz, 1,666.7 Hz and 2,000 Hz. All datasets were recorded either two or three times to obtain error estimates for all data points. ^1^H-^15^N TROSY spectra of Xrn2 and Xrn2 linker (residues 265–293) were recorded with a Bruker Avance III 600-MHz spectrometer equipped with a TXI room temperature probehead.

^19^F spectra were recorded with Bruker Avance III 500-MHz and 600-MHz spectrometers equipped with nitrogen-cooled TCI probeheads. 1D spectra were recorded with the aring pulse sequence from Bruker. ^19^F-CPMG and ^19^F-R_1ρ_ experiments were recorded in a pseudo-2D and pseudo-3D fashion, respectively, using previously published pulse sequences that include an aring sequence^38^. Data were recorded with a recycle delay of 1.0 second. All ^19^F-CPMG experiments were recorded with a constant time T_CPMG_ of 16 ms and CPMG frequencies of 62.6 Hz, 125 Hz, 187.5 Hz, 250 Hz, 312.5 Hz, 375 Hz, 437.5 Hz, 500 Hz, 562.5 Hz, 1,000 Hz, 1,500 Hz, 2,500 Hz and 5,000 Hz (experiments on the 500-MHz spectrometer) and CPMG frequencies of 62.5 Hz, 125 Hz, 187.5 Hz, 250 Hz, 312.5 Hz, 375 Hz, 437.5 Hz, 500 Hz, 625 Hz, 1,000 Hz, 1,500 Hz, 2,000 Hz, 2,500 Hz, 3,000 Hz, 3,500 Hz, 4,000 Hz, 4,500 Hz and 5,000 Hz (experiments on the 600-MHz spectrometer), respectively. ^19^F-R_1ρ_ experiments were recorded with five different spin-lock times T_SL_ (0, 4, 8, 16 and 32 ms) and 18 different spin-lock fields on the 500-MHz spectrometer (50 Hz, 75 Hz, 100 Hz, 125 Hz, 150 Hz, 200 Hz, 250 Hz, 300 Hz, 400 Hz, 500 Hz, 600 Hz, 700 Hz, 800 Hz, 1,000 Hz, 2,500 Hz, 3,000 Hz, 5,000 Hz and 8,000 Hz) as well as 18 different spin-lock fields on the 600-MHz spectrometer (50 Hz, 75 Hz, 100 Hz, 125 Hz, 150 Hz, 200 Hz, 250 Hz, 300 Hz, 400 Hz, 500 Hz, 600 Hz, 800 Hz, 1,000 Hz, 1,500 Hz, 2,000 Hz, 3,000 Hz, 5,000 Hz and 8,000 Hz). The ^19^F frequency carrier was centered on the respective peak maximum in the 1D ^19^F spectrum. All ^19^F relaxation dispersion datasets were recorded in triplicate to obtain experimental errors for all data points, with the exception of V416C (Supplementary Fig. 14), where the errors in the intensities were determined based on the noise level of the spectra. NMR spectra were processed with the NMRPipe/NMRDraw software suite^56^.

### NMR structure calculations of the ZnF

All spectra used for the structure calculation were acquired at 288 K on Bruker Avance III 500-MHz and 600-MHz spectrometers equipped with nitrogen-cooled TCI probeheads. Assignment of the ZnF (residues 265–293) backbone and sidechain resonances was obtained based on the following spectra: ^1^H^15^N-HSQC, ^1^H^13^C-HSQC, 3D-HNCACB, 3D-HN(CA)CO, 3D-H(CC)(CO)NH-TOCSY and 3D-(H)CC(CO)NH-TOCSY. All assignment spectra were processed using TopSpin 4.0.2 and analyzed with CARA. Distance restraints for the structure calculation were obtained from 3D ^15^N NOESY-HSQC (mixing time 150 ms), 3D ^13^C NOESY-HSQC (mixing time 150 ms) and 2D ^13^C_aro_-NOESY-HSQC spectra with automatic peak picking using NMRFAM-SPARKY^57^. Torsion angle restraints were derived from chemical shift assignments using the TALOS-N web server^58^. Additional restraints were manually added for Zn^2+^ coordination, where the Zn^2+^ ion was included using a CYSZ residue. A structure bundle was calculated using the automated NOE assignment protocol implemented in CYANA, where seven iterations were performed with 100 initial and 20 final structures^59^. NMR assignments are deposited in the Biological Magnetic Resonance Data Bank (BMRB) under accession number 50997, and the NMR structure is deposited in the PDB under accession number 7PVM.

### Titration of substrates

HMQC spectra of 50 μM Xrn2 with different substrates were recorded at 313 K in the presence of 5 mM MgCl_2_ unless indicated otherwise. 3′,5′-bisphosphoadenosine (100 mM stock solution) was added to a final concentration of 500 μM. 5′-phosphorylated A5-DNA (2 mM stock solution) was added to a final concentration of 200 μM. xrRNA prepared by IVT was added to a final concentration of 100 μM.

### Fluorescence anisotropy

Fluorescence anisotropy experiments were performed in 25 mM HEPES (pH 7.3), 125 mM NaCl and 0.002% Triton with fluorescein-labeled 5-mer 5′-GGAGU^Fsc^-3′ and 10-mer 5′-GGAGGAGAGU^Fsc^-3′ RNAs (where U^Fsc^ is a thiouridin covalently labeled with iodacetamido-fluorescein) generated as described above. RNA concentrations were 5 nM (5-mer RNA) or 1 nM (10-mer RNA); protein concentrations were 0, 5, 10, 30, 100, 400 and 1,000 nM (5-mer RNA) and 0, 2, 5, 10, 30, 80 and 200 nM (10-mer RNA). Data were acquired with a TECAN Spark microplate reader with excitation and emission wavelengths of 485 nm and 535 nm, respectively. Fluorescence anisotropy data were analyzed with in-house-written MATLAB scripts and fitted to a one-site binding model according to

$$r_{obs} = r_{min} + s \ast \left( {\frac{{\left[ {RNA} \right] + \left[ P \right] + K_D}}{2} - \sqrt {\left( {\frac{{\left[ {RNA} \right] + \left[ P \right] + K_D}}{2}} \right)^2 - \left[ {RNA} \right]\left[ P \right]} } \right)$$

where r_obs_ is the observed anisotropy, r_min_ is the minimum anisotropy, s is a scaling factor, [RNA] is the concentration of fluorescein-labeled RNA, [P] is the protein concentration and K_D_ is the dissociation constant. The binding experiments were recorded in triplicate; the error (standard deviation) is based on the three measurements. The source data can be found online.

### Data processing, analysis and fitting

Methyl-TROSY CPMG pseudo-3D data were processed with NMRPipe^56^ and analyzed with in-house-written MATLAB scripts. In brief, peak intensities I_υ_ corresponding to CPMG frequencies υ_CPMG_ were extracted from 2D planes with NMRPipe and normalized to a reference intensity I_0_ obtained by omitting the CPMG element. The normalized intensity I_υ_/I_0_ was then converted into effective transverse relaxation rates R_2,eff_ by assuming an exponential decay. The data were fitted numerically to a global two-state model to extract the exchange parameters p_GS_, k_ex_, Δω_C_^i^ and Δω_H_^i^, R_2,Mq_^i^ and R_2,SQ_^i^ where the index i refers to the residue number. A minimum standard deviation of 0.2 Hz was assumed for the fit.

^19^F CPMG pseudo-2D datasets were processed with NMRPipe^56^ and analyzed with in-house-written MATLAB scripts as described previously^38^. Minimum standard deviations were set to 2 Hz to avoid excessive weighting of data points with accidentally low errors.

On-resonance R_1ρ_ pseudo-3D datasets were processed with NMRPipe, and R_1ρ_ rates were extracted from the exponential fit of resonance intensities from different spin-lock times T_SL_. R_1ρ_ data were fitted numerically. To avoid errors arising from approximations, the shift of the average peak was calculated as the imaginary part of the complex eigenvalues of the exchange matrix during parameter optimization. Minimum standard deviations were set to 2 Hz to avoid excessive weighting of data points with accidentally low errors.

To extract errors in the parameters extracted from the NMR relaxation data, 500 cycles of Monte Carlo simulations were carried out by random variation of R_2_ rates according to their standard deviation and subsequent execution of the numerical fitting routine. Reduced χ^2^ (χ^2^_υ_) surfaces were calculated by constrained fits, in which the probed parameter (k_ex_, p_GS_ or | Δω |) was systematically fixed for a range of values while all other parameters were varied to minimize the value of χ^2^_υ_.

The helical wheel in Fig. 3 was generated with DrawCoil 1.0 (https://grigoryanlab.org/drawcoil/). All pictures of structures were prepared with ChimeraX^60^, and all pictures of 2D NMR spectra were prepared using NMRView (www.onemoonscientific.com). The sequence alignment in Supplementary Fig. 8 was generated with ClustalX 2.1.

### Reporting summary

Further information on research design is available in the Nature Research Reporting Summary linked to this article.

## Online content

Any methods, additional references, Nature Research reporting summaries, source data, extended data, supplementary information, acknowledgements, peer review information; details of author contributions and competing interests; and statements of data and code availability are available at 10.1038/s41589-022-01111-6.

## Supplementary information

Supplementary Information Supplementary Figs. 1–21 and Supplementary Tables 1–8.

Reporting Summary

## Data Availability

The structure factors and coordinates for the Xrn2 core protein have been deposited in the PDB under accession code 7OPK. The assignments for the Xrn2 ZnF have been deposited in the BMRB under accession number 50997. The atomic coordinates for the Xrn2 ZnF have been deposited in the PDB under accession code 7PVM. All other relevant data are available in the Source Data provided with this paper and are also available upon reasonable request to the corresponding author.

## Source data

Source Data Fig. 1 RNA binding data and RNA degradation data.
Source Data Fig. 5 RNA degradation data.
Source Data Extended Data Fig. 6 RNA degradation data.
Source Data Extended Data Fig. 9 RNA binding data.
