# Supplementary material for: Observation of conformational changes that underlie the catalytic cycle of Xrn2
Source: Nat Chem Biol. 2022 Aug 25;18(10):1152–60. doi: 10.1038/s41589-022-01111-6 (PMC9512700; doi:10.1038/s41589-022-01111-6)
Supplement: Supplementary file 1 — Supplementary Figs. 1–21 and Supplementary Tables 1–8. [file 41589_2022_1111_MOESM1_ESM.pdf]

nature portfolio

https://doi.org/10.1038/s41589-022-01111-6

Supplementary information

Observation of conformational changes that underlie the catalytic cycle of Xrn2

In the format provided by the authors and unedited

Supplementary Information for

Observation of conformational changes that underlie the catalytic cycle of Xrn2

Jan H. Overbeck[1], David Stelzig[1], Anna-Lisa Fuchs[1,2], Jan Philip Wurm[1] & Remco Sprangers[1,*]

1: Institute of Biophysics and Physical Biochemistry, Regensburg Center for Biochemistry, University of Regensburg, 93053 Regensburg, Germany.
2: Current address: TU Munich, Department of Informatics, Boltzmannstr. 3, Garching, Germany.

*Corresponding Author:
E-mail: remco.sprangers@ur.de

Table of contents

Supplementary Fig. 1 | Structure of Xrn1 S2
Supplementary Fig. 2 | Model of the translocation-hydrolysis mechanism in Xrn1/Xrn2 S3
Supplementary Fig. 3 | Comparison of our crystal structure of CtXrn2 with other structures S4
Supplementary Fig. 4 | Comparison of the His-Trp stacking sandwich S5
Supplementary Fig. 5 | NMR structural bundle of the Xrn2 zinc finger (ZnF) S6
Supplementary Fig. 6 | Comparison of the Xrn2 ZnF with known CCHC ZnF structures S7
Supplementary Fig. 7 | HPLC-based Xrn2 activity assays S8
Supplementary Fig. 8 | Sequence alignments of Xrn1 and Xrn2 homologues S9
Supplementary Fig. 9 | 13C SQ and 1H-13C MQ CPMG RD data of Xrn2 at 313 K S10
Supplementary Fig. 10 | 13C SQ and 1H-13C MQ CPMG RD data of of remotely located residues S11
Supplementary Fig. 11 | Initial 19F spectra of BTFA-labeled Xrn2 1-875 and Xrn2 1-875 N12C S12
Supplementary Fig. 12 | Reduced χ2 surfaces of 19F RD data S13
Supplementary Fig. 13 | Monte Carlo trials of 19F CPMG and R1ρ relaxation dispersion data S14
Supplementary Fig. 14 | 19F CPMG profiles of additional BTFA-labeled Xrn2 cysteine mutants S15
Supplementary Fig. 15 | 1H spectra of pdA5 degradation by Xrn2 S16
Supplementary Fig. 16 | pAp and pdA2 induce the same global conformational change in Xrn2 S17
Supplementary Fig. 17 | Xrn2 only degrades the unstructured 5'-region of the xrRNA S18
Supplementary Fig. 18 | Intensity changes in Xrn2 Ileδ1-methyl groups upon addition of xrRNA S19
Supplementary Fig. 19 | The PBS (PAXT-1 – binding segment) of CtXrn2 is folded S20
Supplementary Fig. 20 | Xrn2 and Rai1 from Chaetomium thermophilum form a stable complex S21
Supplementary Fig. 21 | CtRai1 does not degrade RNA and does not enhance the activity of CtXrn2 S22
Supplementary Table 1 | Protein constructs used in this study S23
Supplementary Table 2 | Constructs used for resonance assignments S24
Supplementary Table 3 | Rates of RNA degradation by Xrn2 S26
Supplementary Table 4 | RNA sequences S27
Supplementary Table 5 | Point mutants of the Xrn2 ΔZnF N12C construct S28
Supplementary Table 6 | Fit parameters as obtained by Monte Carlo trials of 19F data S29
Supplementary Table 7 | Crystallography: Data collection and refinement statistics S30
Supplementary Table 8 | Structural statistics for the NMR solution structure of the zinc finger S31
Supplementary References S32

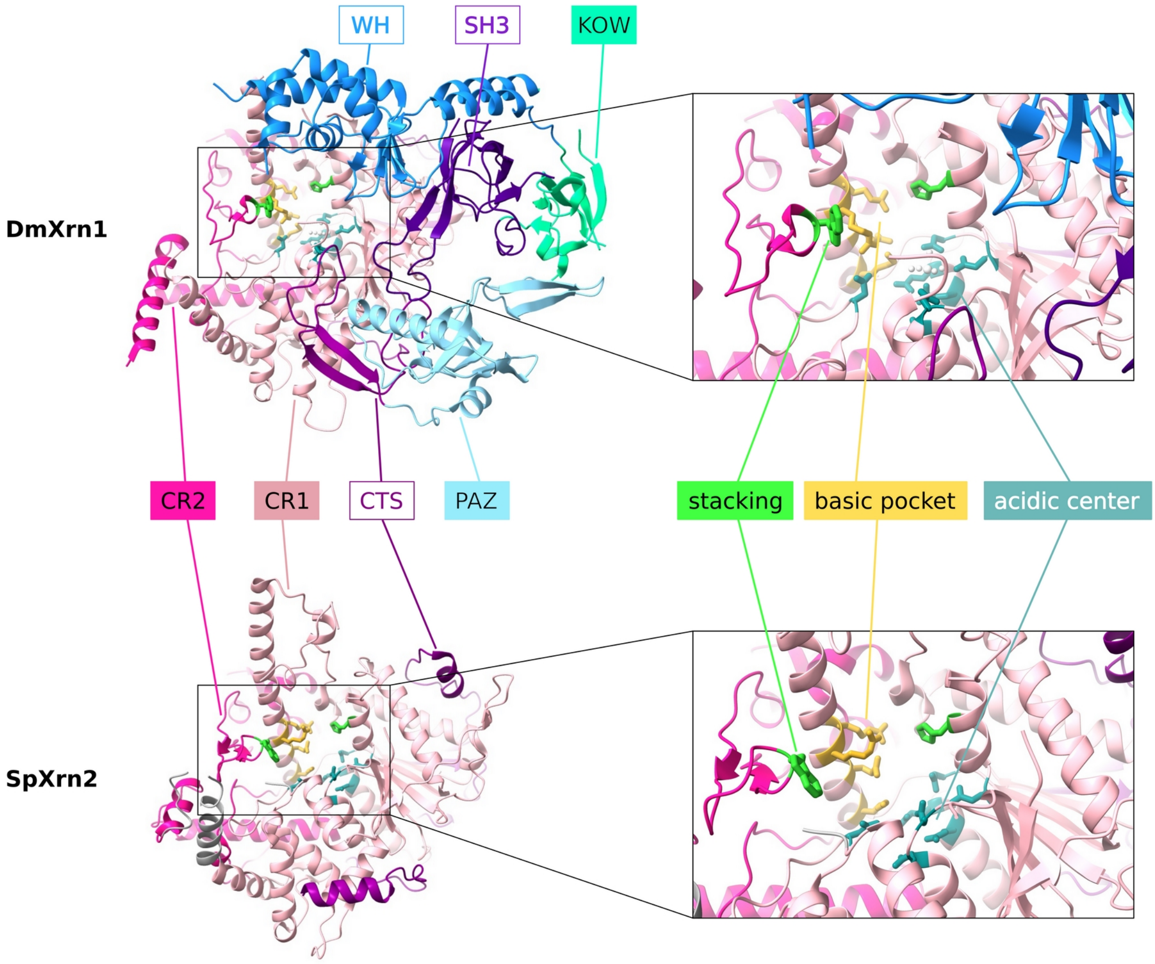

Supplementary Fig. 1 | Structure of Xrn1 from *D. melanogaster* (DmXrn1; PDB: 2Y35 (1)) and Xrn2 from *S. pombe* (SpXrn2; PDB: 3FQD (2)) with highlighted domains and features
Xrn1 and Xrn2 share a conserved exoribonuclease core formed by the conserved regions 1 (CR1, salmon) and 2 (CR2, pink) and a less-conserved C-terminal segment (CTS, purple). In addition, Xrn1 features a folded C-terminal region composed of a PAZ domain (cyan), a Kyprides-Ouzounis-Woese (KOW) domain (lime), an SH3 domain (dark purple) and a winged helix (WH) domain (blue). The C-terminal regions of Xrn1 and Xrn2 are disordered, poorly conserved and not displayed here. In the active site (insets), both Xrn1 and Xrn2 have a conserved acidic magnesium binding site, a basic pocket that recognizes the monophosphorylated 5'-end of the RNA substrate (yellow) as well as a conserved His-Trp pair which sandwiches the first three bases of the substrate by aromatic stacking (green).

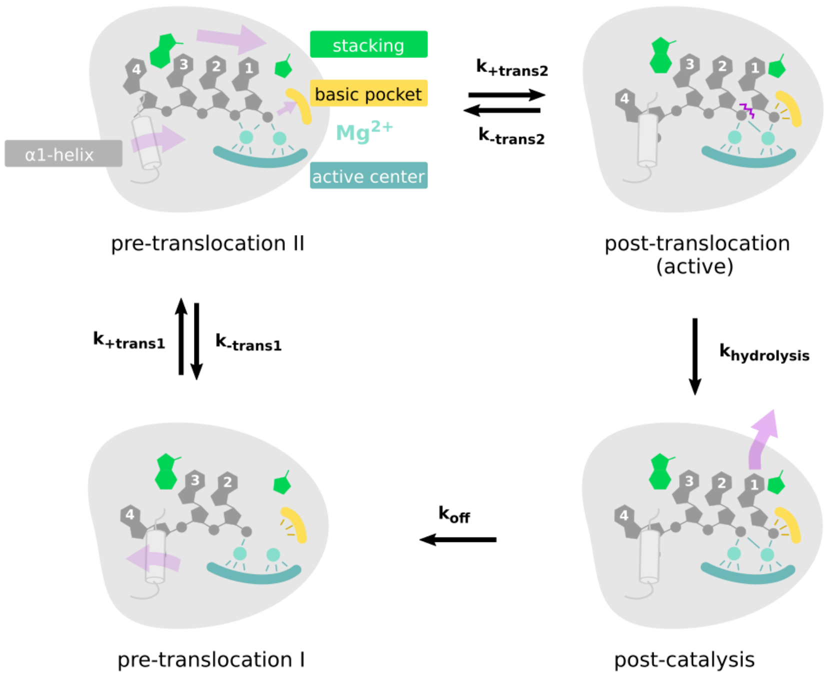

Supplementary Fig. 2 | Model of the translocation-hydrolysis mechanism in Xrn1/Xrn2

The enzyme interacts with the RNA substrate (dark grey), in which the first three nucleotides are stacked between the conserved His/Trp residues (green; top left; pre-translocation II). Due to a conformation change (k_{trans2}) the RNA is translocated to a hydrolysis-prone (active) position (post-translocation; top right), where hydrolysis can take place. After hydrolysis ($k_{hydrolysis}$) the bond between the terminal 5' nucleotide and the RNA body is cleaved (post-catalysis; bottom right). Subsequently, the cleaved 5' nucleoside monophosphate is released (k_{off}; pre-translocation I; bottom left). Finally, the conserved tryptophan residue facilitates the motion of the next base into the aromatic stack (green) and brings the enzyme substrate complex again into the pre-translocation II (active) position (top left). Structures of the Xrn enzyme in the pre-translocation state II (Xrn1 in the complex with mRNA within the 80S ribosome-Xrn1 complex (3)) as well as in the post-translocation state (Xrn1 in complex with a 5′-phosphorylated 11-mer DNA oligonucleotide (4)) have been solved. The N-terminal α1-helix has been suggested to function as a brownian ratchet in the substrate translocation process.

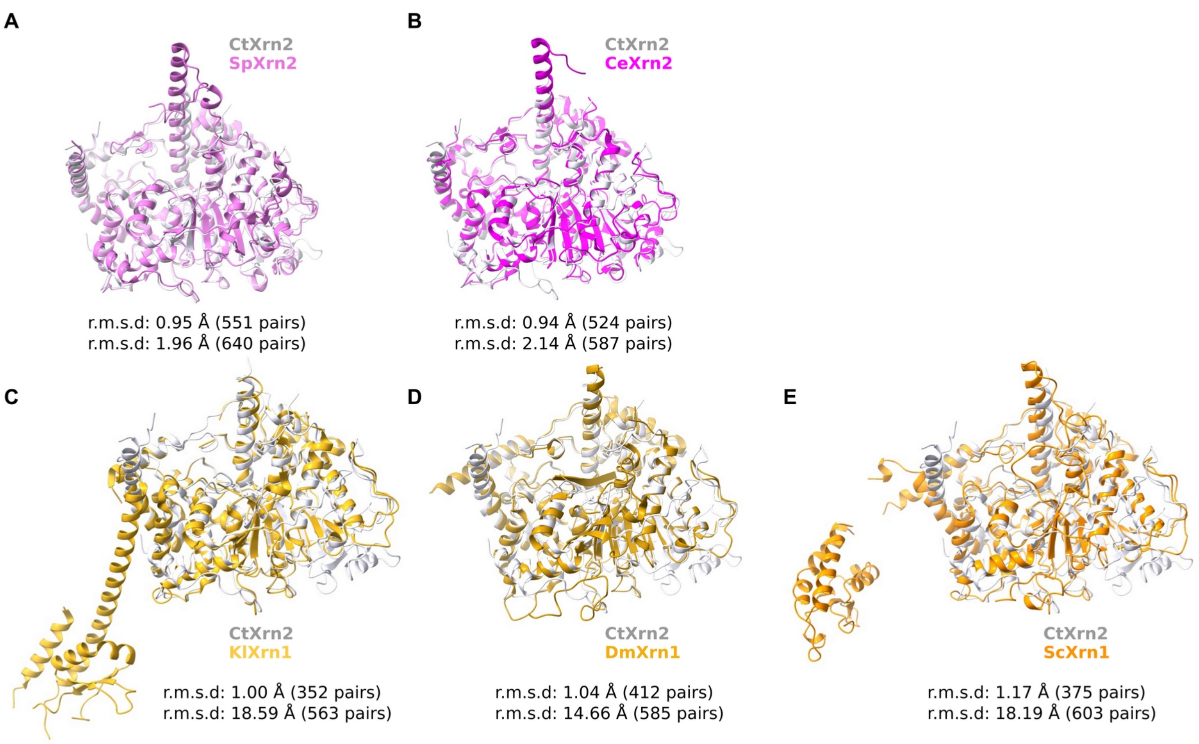

Supplementary Fig. 3 | Comparison of our crystal structure of CtXrn2 with other structures of 5'-3' exoribonucleases

Overlay of the CtXrn2 structure solved in this study with (A) SpXrn2 (PDB: 3FQD (2)), (B) CeXrn2 (PDB: 5FIR (5)), (C) KlXrn1 (PDB: 3PIE (6)), (D) DmXrn1 (PDB: 2Y35 (4)) and (E) ScXrn1 (PDB: 6Q8Y (3)). The r.m.s.d. values of matched Cα atom pairs (bottom) and a pruned selection thereof (top, as obtained by the ChimeraX match command) are indicated. For clarity, the C-terminal PAZ, KOW, SH3 and WH domains are omitted from the Xrn1 structures.

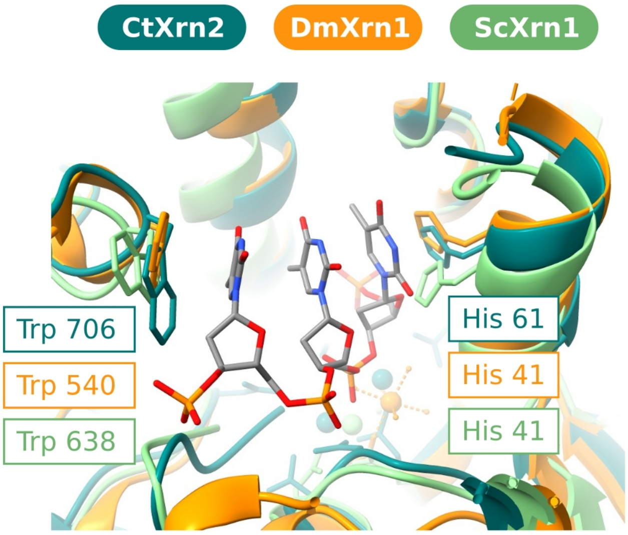

Supplementary Fig. 4 | Comparison of the His-Trp stacking sandwich

In the CtXrn2 structure solved in this study (dark green), the His-Trp pair (His61-Trp706) is similarly positioned as in the DmXrn1 structure (orange, PDB 2Y35, shown with the DNA pseudosubstrate) and the ScXrn1 structure (light green, PDB 6Q8Y, shown without the RNA substrate for clarity).

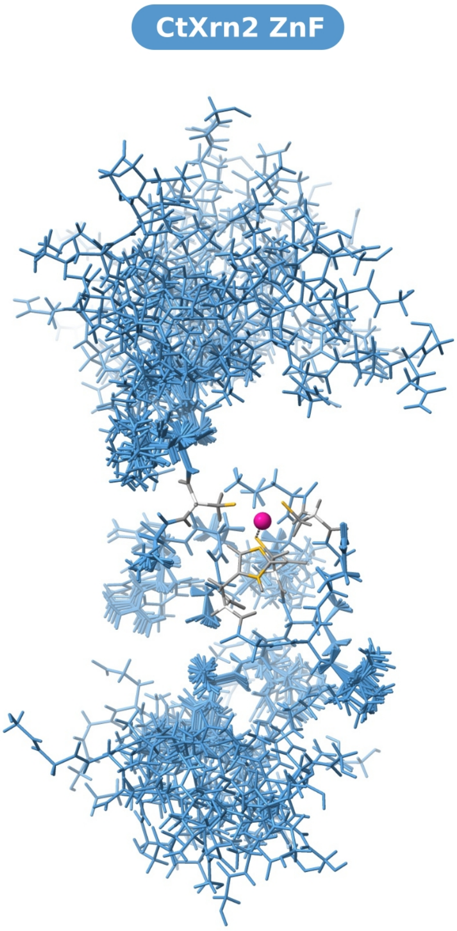

Supplementary Fig. 5 | NMR structural bundle of the Xrn2 zinc finger (ZnF)

Shown are the 20 lowest energy structures from the structure calculation. The Zn^{2+} ion is shown in pink, the coordinating CCHC residues are shown in grey/yellow. The core of the ZnF is well defined, the regions N- and C- terminal to the ZnF are highly flexible, which ensures that the ZnF can be placed into the loop in the Xrn2 core.

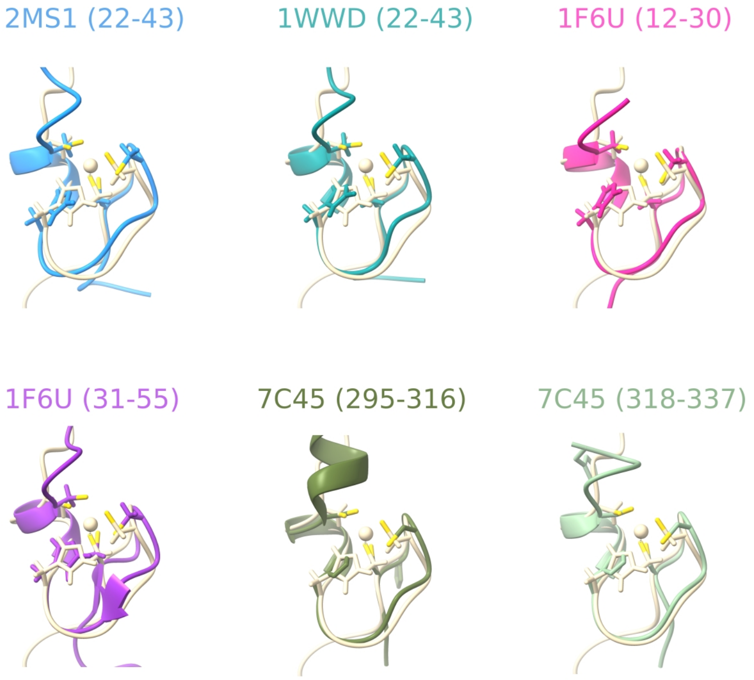

Supplementary Fig. 6 | Comparison of the Xrn2 ZnF with known CCHC ZnF structures

The ZnF of Xrn2 adopts the same fold as other known CCHC ZnF, exemplified here by zinc fingers from the nucleocapsid protein of MLV (PDB 2MS1 (7), 1WWD (8)), from the nucleocapsid protein of HIV-1 (PDB 1F6U (9)) and from RNase D (PDB 7C45 (10)). The structure of the ZnF that we determined here is shown in yellow.

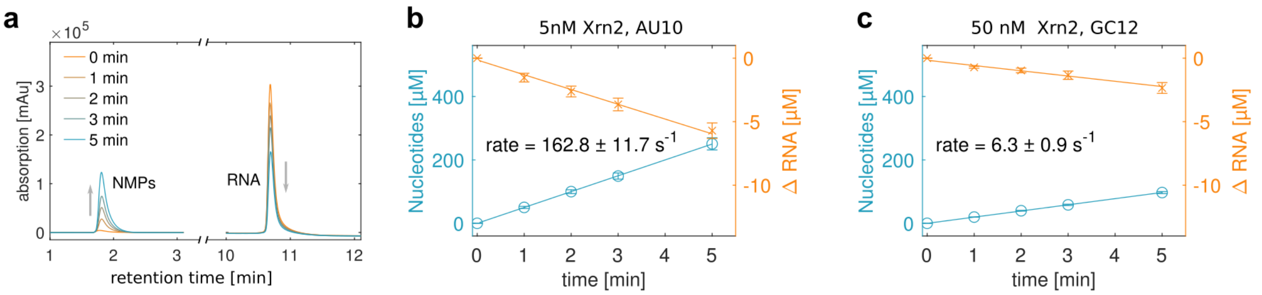

Supplementary Fig. 7 | HPLC-based Xrn2 activity assays

(a) For each time point in the activity assay substrate (RNA) and products (NMPs) (see Supplementary methods) are separated on an anion-exchange HPLC column. HPLC peak areas are quantified and are converted into nucleotide monophosphates and RNA concentrations based on their standard calibration curves. The changes in substrate and product concentration are simultaneously fit to a linear degradation model (b,c). The slope of these curves are converted into turnover numbers based on the used enzyme concentration. Data points are shown as mean ± s.d., as derived from 2 independent degradation series.

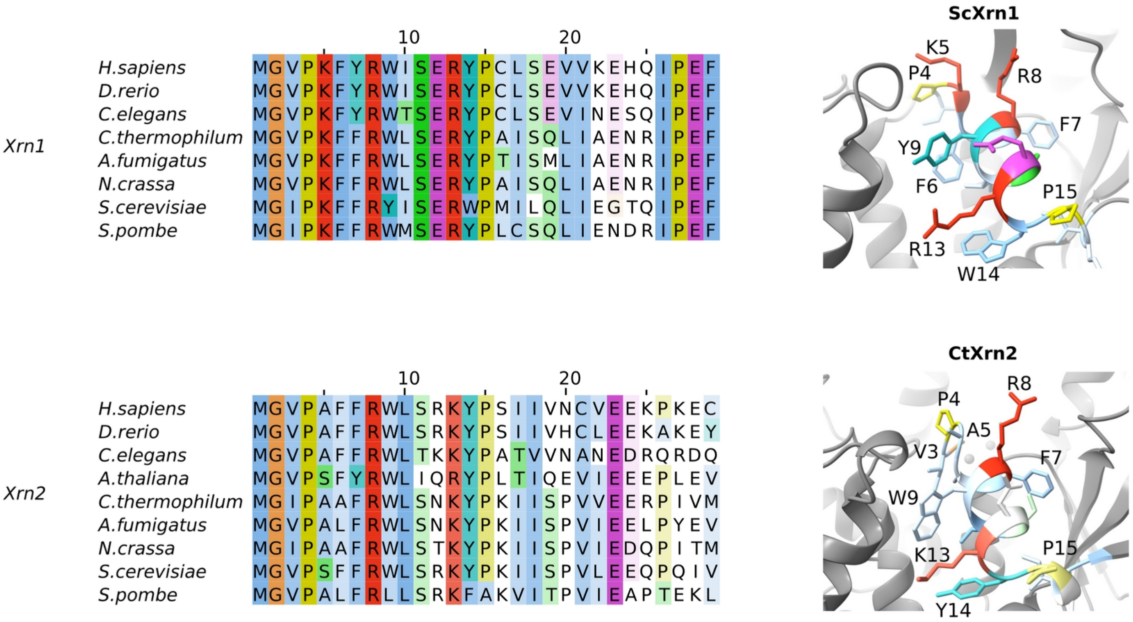

Supplementary Fig. 8 | Sequence alignments of Xrn1 and Xrn2 homologues and close-up view of the α1-helix in the ScXrn1 (pre-translocation state II) and CtXrn2 (apo) structures

The N-termini of Xrn1 and Xrn2 are conserved from lower to higher eukaryotes (from *S. pombe* and *S. cerevisiae* to *D. rerio* and *H. sapiens*) and are similar to each other in sequence and structure. The only notable differences include the presence of a positively charged residue (Lys) at position 5 and a negatively charged residue (Glu) at position 12 in the sequence of Xrn1.

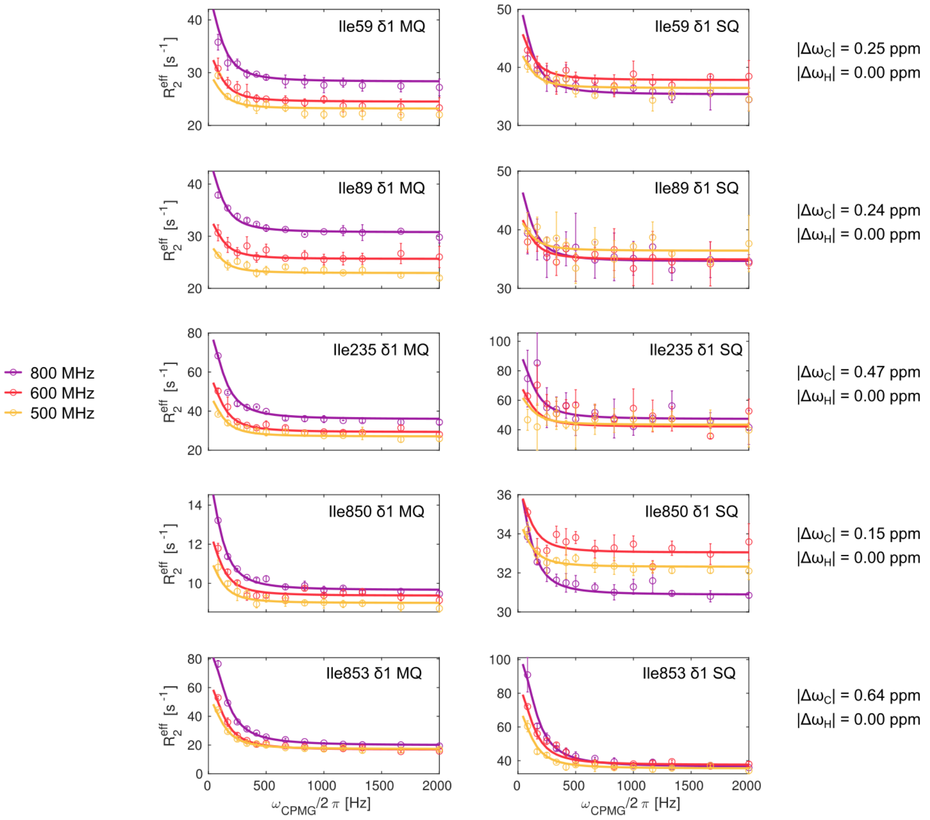

Supplementary Fig. 9 | ^{13}C SQ and ^{1}H-^{13}C MQ CPMG RD data of Xrn2 at 313 K

The largest chemical shift differences between ground state and excited state were obtained for Ile853 ($|\Delta\omega_C|$ = 0.64 ppm) and Ile235 ($|\Delta\omega_C|$ = 0.47 ppm). For those residues the relative values of $\Delta\omega$ and k_{ex}, that define the NMR timescale, are comparable in size and place the motion in the intermediate exchange regime. It is generally possible to extract information on the populations and the chemical shift differences, when the dynamics are not in the fast exchange limit. However, when we applied a Monte Carlo procedure to extract errors for the fitting parameters, we found a considerable spread in the solution space, with a characteristic interdependence of p_{GS} and the chemical shift differences (**SI Appendix, Fig. S13**) that is given by a residue-dependent constant $\Phi_{ex} = p_{GS}*p_{ES}*\Delta\omega^2$. Of note, even when the lower limit of $|\Delta\omega| = 2*\ \Phi_{ex}^{1/2}$ is considered, the exchange still remains in the intermediate regime for Ile853 ($p_{GS}*p_{ES} \leq 0.25$). The extracted fitting parameters, including errors are summarized in Supplementary Table 6. Data points are shown as mean ± s.d., as derived from at least 2 duplicate NMR measurements.

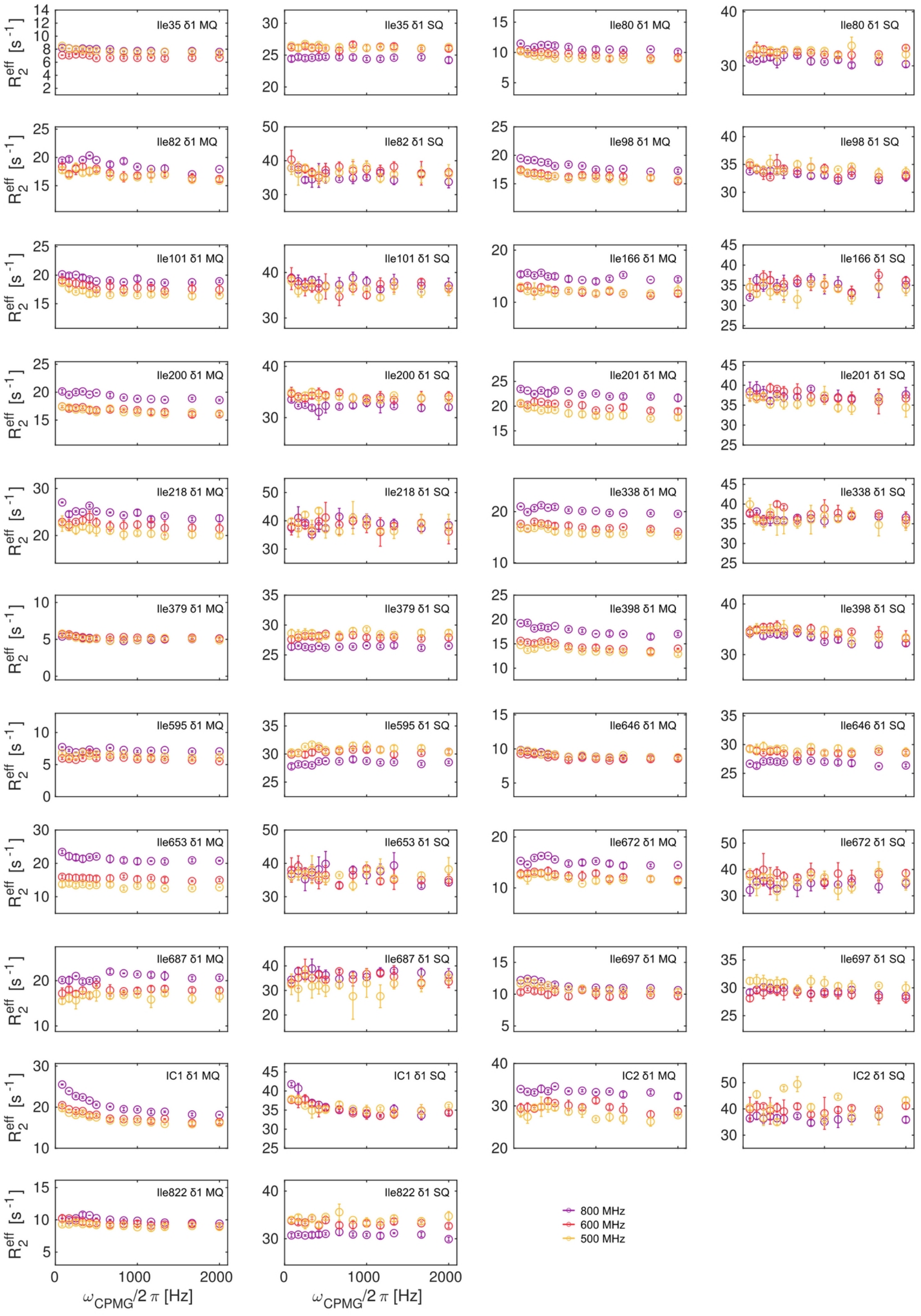

Supplementary Fig. 10 | ^{13}C SQ and ^{1}H-^{13}C MQ CPMG RD data of Xrn2 at 313 K for residues not included in the fit

Methyl groups of residues that are not located close to the N-terminal α1-helix give rise to flat relaxation dispersion profiles. We attribute the global lack of dispersions to the fact, that the structural changes that we detect are spatially confined to the region around the α1-helix. An exception is observed for the isoleucine cluster peak 1 (IC1), for which relaxation rates are increased at lower CPMG frequencies in all datasets. Since this residue is located on the rear side of the protein, we attribute this to an unrelated motion. Data points are shown as mean ± s.d., as derived from at least 2 duplicate NMR measurements.

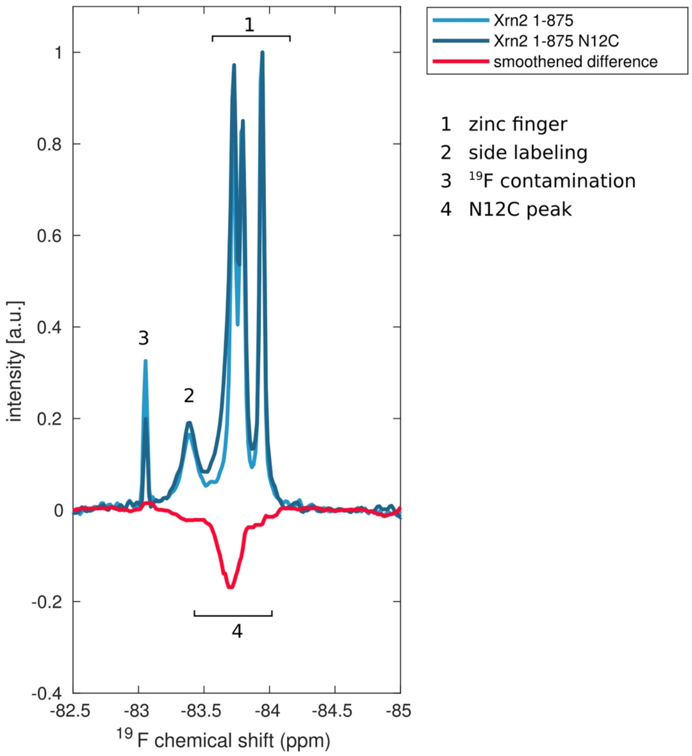

Supplementary Fig. 11 | Initial ^{19}F spectra of BTFA-labeled Xrn2 1-875 and Xrn2 1-875 N12C
We labeled both wild type Xrn2 as well as the Xrn2 N12C mutant for 30 minutes at 30ºC. Both spectra show three strong peaks (labeled “1”) between between -83.7 and -84.0 ppm, which we attributed to the three zinc finger cysteine residues in the loop between residues 265 and 295, as well as a broad peak at -83.4 ppm (labeled “2”) and a sharp peak at -83.0 ppm (labeled “3”), that likely come from the labeling of other cysteine residues in Xrn2 (e.g. Cys 63, 184, 345 and 620). A difference spectrum (Spectrum Xrn2 1-875 minus spectrum Xrn2 1-875 N12C) revealed a clear additional peak well above the noise level around -83.7 ppm, which we attributed to the ^{19}F labeled additional cysteine at position 12 (labeled “4”). CPMG spectra of both samples showed the distinct presence of relaxation dispersion in the N12C sample. To be able to accurately quantify those dispersion we removed the ZnF from the protein (to eliminate the resonances labeled “1”), reduced the labeling temperature to 4ºC (to eliminate the resonance labeled “2”) and purified the labeled enzyme via size exclusion chromatography (to eliminate the resonance labeled “4”). The resulting ^{19}F spectrum shows a single resonance at -83.78 ppm (**Fig. 3C**).

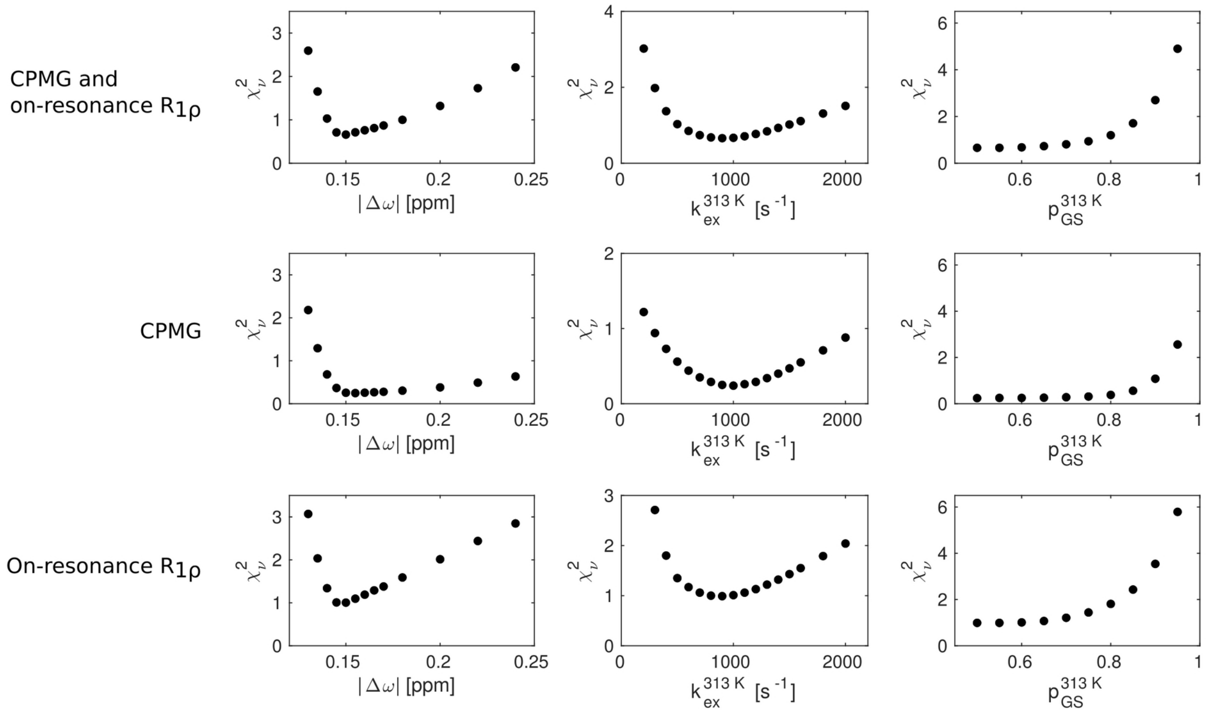

Supplementary Fig. 12 | Reduced χ² surfaces obtained from constrained parameter optimization of ¹⁹F RD data

One dimensional reduced χ^2 surfaces for the simultaneous analysis of ^{19}F CPMG and $R_{1\rho}$ relaxation data (top), or independent analysis of ^{19}F CPMG data (middle) or $R_{1\rho}$ data (bottom). For each analysis, reduced χ^2 values were obtained as a function of the chemical shift difference (left), the exchange rate at 313 K (middle) or the ground state population at 313 K (right) after optimization of all other fitting parameters. The data displays defined minima for $|\Delta\omega|$ and k_{ex}^{313K}, while the populations p_{GS}^{313K} are not well defined; the product $\Phi_{ex} = p_{GS}*p_{ES}*|\Delta\omega|^2$ does changes by much for $0.5 < p_{GS}^{313K} < 0.8$. Note that the upper bound of p_{GS} is defined more precisely than the lower bound; the reduced χ^2 value increases rapidly for $p_{GS} > \sim 0.8$. The extracted fitting parameters, including errors are summarized in Supplementary Table 6.

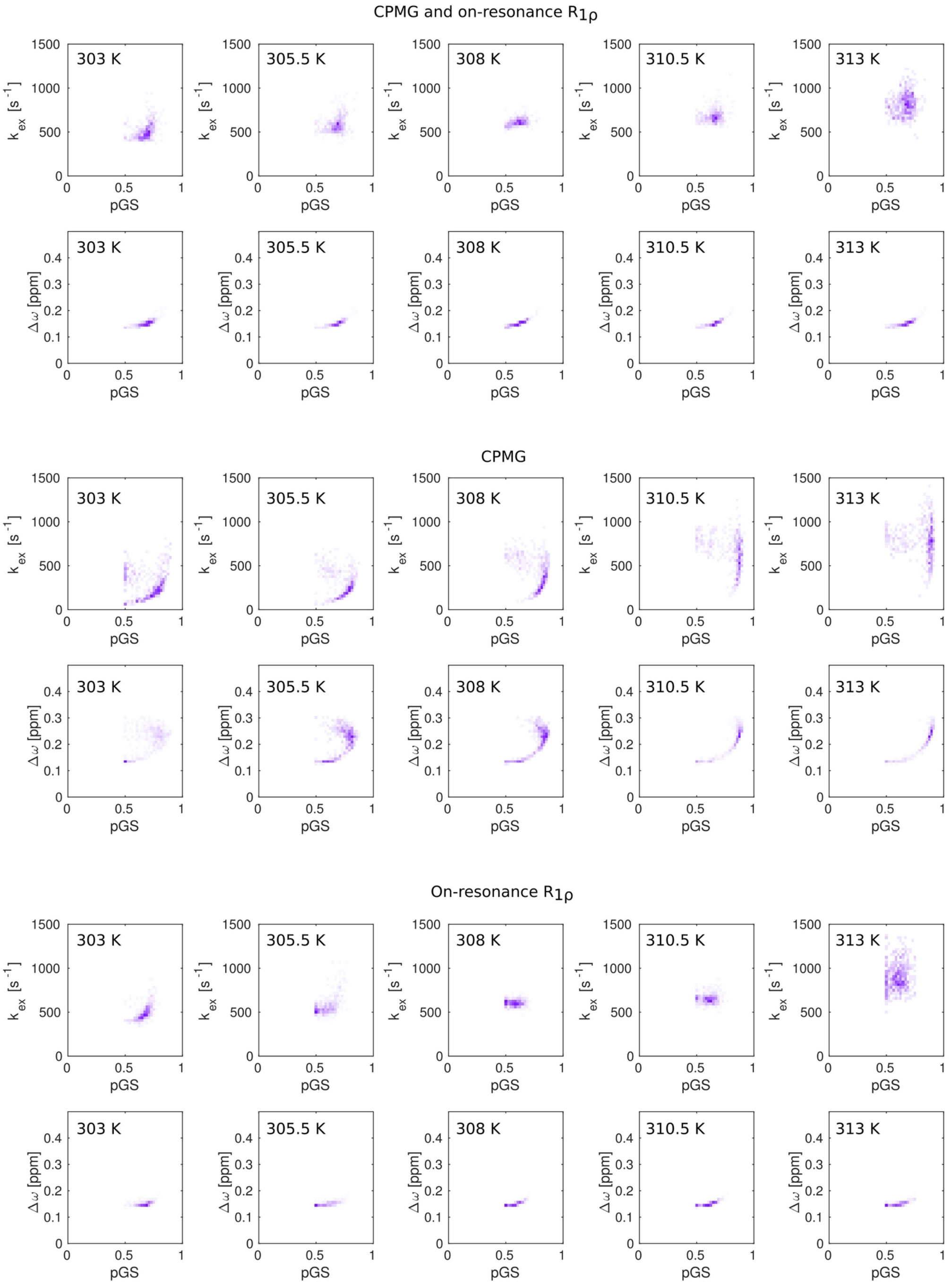

Supplementary Fig. 13 | Monte Carlo trials of ^{19}F CPMG and $R_{1\rho}$ relaxation dispersion data

Distribution of fit parameters obtained from MC simulations, where ^{19}F CPMG and $R_{1\rho}$ relaxation data were fitted simultaneously (top), or where either the ^{19}F CPMG (middle) or $R_{1\rho}$ relaxation data (bottom) were fitted independently. Note the typical correlation between Δω and p_{GS} at all temperatures. This is especially prominent in the fits of the CPMG experiments only, that are not able to define the exchange parameters with high precision. The extracted fitting parameters, including errors are summarized in Supplementary Table 6.

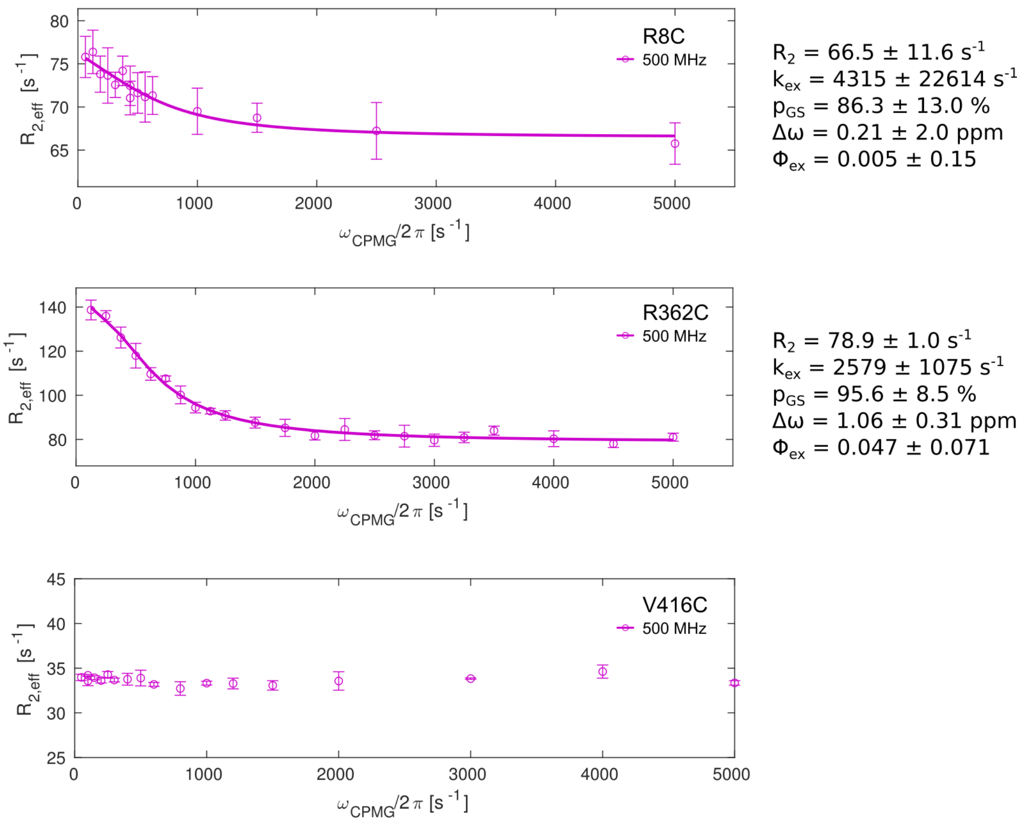

Supplementary Fig. 14 | ^{19}F CPMG profiles of additional BTFA-labeled Xrn2 cysteine mutants

Relaxation dispersions were observed for ^{19}F probes attached to artificially introduced cysteine residues in (R8C) or next to (R362C) the α1-helix, but not at a more distant labeling site (V416C). Note that the fitting parameters are not well defined as only CPMG data (and no $R_{1\rho}$ data) at one temperature and one field strength has been recorded. These data, however, clearly indicate that the motions in the N-terminal region can be detected independently of the exact location of the ^{19}F label in or around this helix. Data points are shown as mean ± s.d., as derived from 3 duplicate NMR measurements (R8C and V362C). For V416 the errors in the intensities were determined based on the noise level of the NMR spectra.

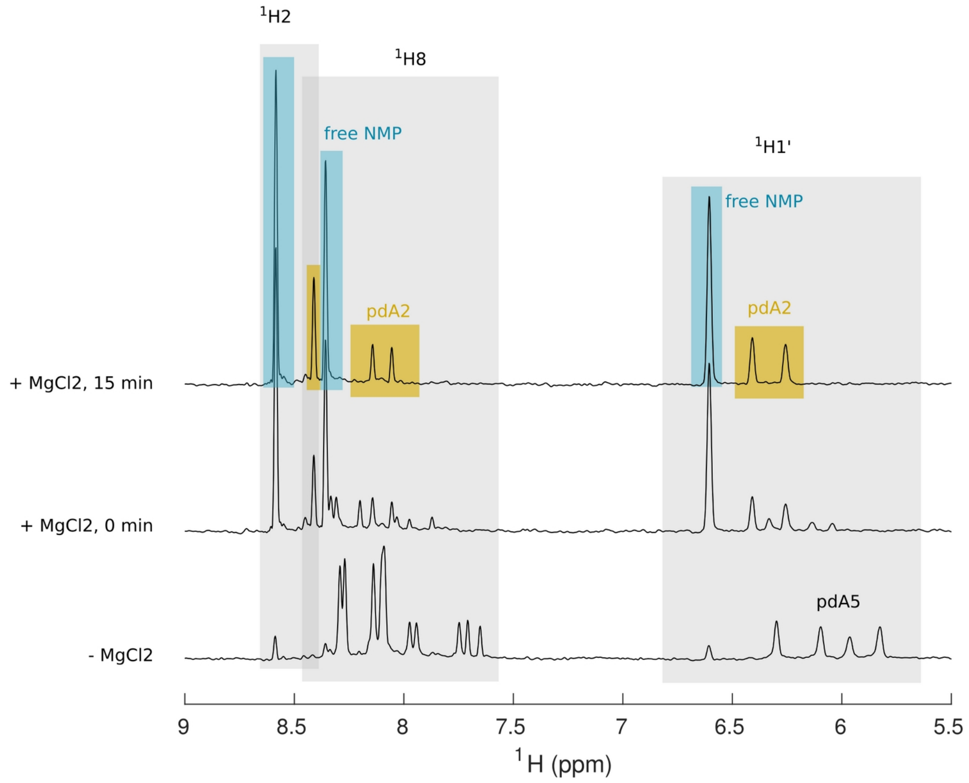

Supplementary Fig. 15 | ^{1}H spectra of pdA5 degradation by Xrn2

200 µM pdA5 is added to 50 µM Xrn2 in the absence of catalytically important magnesium ions (bottom spectrum). The H1' sugar proton resonances in pdA5 are clearly visible around 6 ppm, showing that pdA5 is not degraded by Xrn2 under these conditions. Upon addition of magnesium the turnover of pdA5 into pdA2 and free nucleotides is initiated (middle spectrum). Within 15 minutes, the pdA5 is completely turned over into pdA2 (top spectrum).

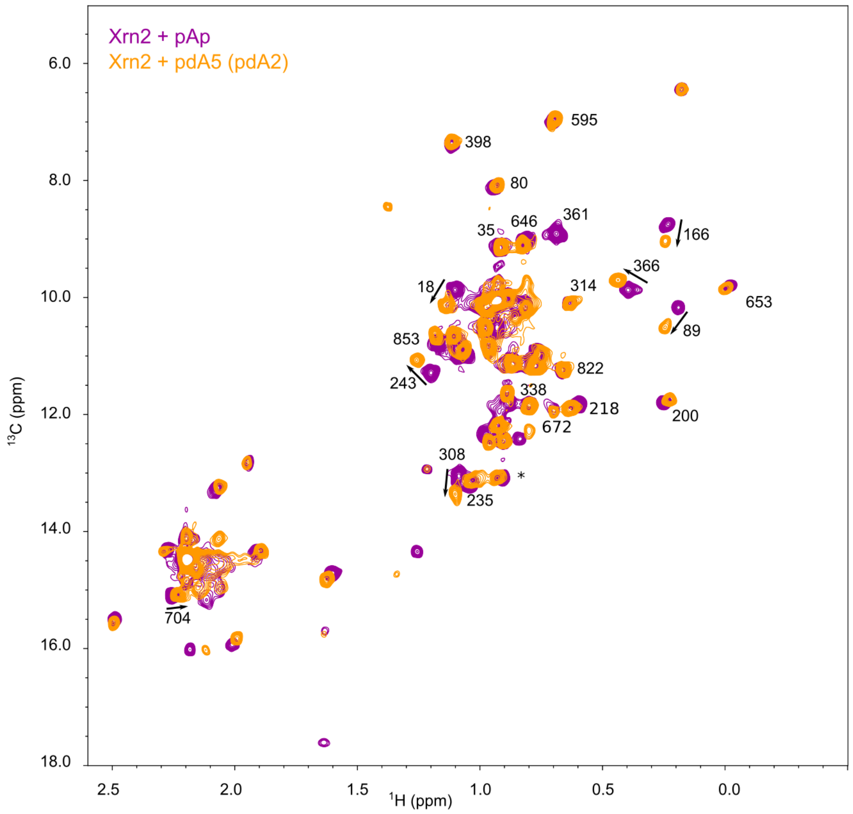

Supplementary Fig. 16 | pAp and pdA2 induce the same global conformational change in Xrn2

Overlay of the ^{1}H-^{13}C HMQC methyl-TROSY spectra in the presence of pAp (purple) and pdA2 (orange, produced in the NMR tube from pdA5), recorded at 18.8 T and 313 K. Resonances of residues that display a different chemical shift in the pAp- and pdA2-bound states are labeled.

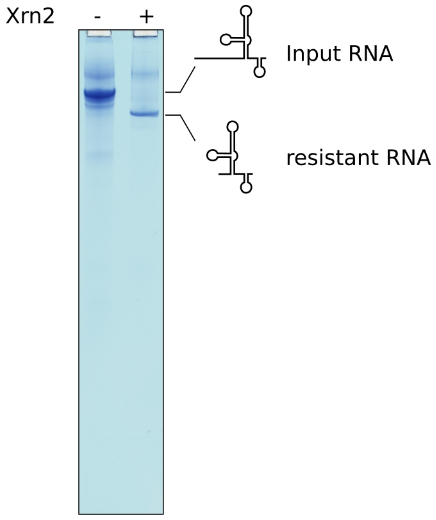

Supplementary Fig. 17 | Xrn2 degrades the unstructured 5’-region of the xrRNA until it encounters the degradation-resistant pseudoknot structure

The substrate RNA (Table S4) carries a single stranded region at the 5’-end, followed by the pseudoknot structure. The linear 5’-end of the RNA is rapidly degraded, but further degradation is sterically blocked by the pseudoknot, giving rise to an Xrn2-resistant RNA fragment that can be detected by urea polyacrylamide gel electrophoresis. Note that this resistant fragment is stable also during NMR measurement over several hours. This experiment was performed once and the results were confirmed in independent NMR experiments.

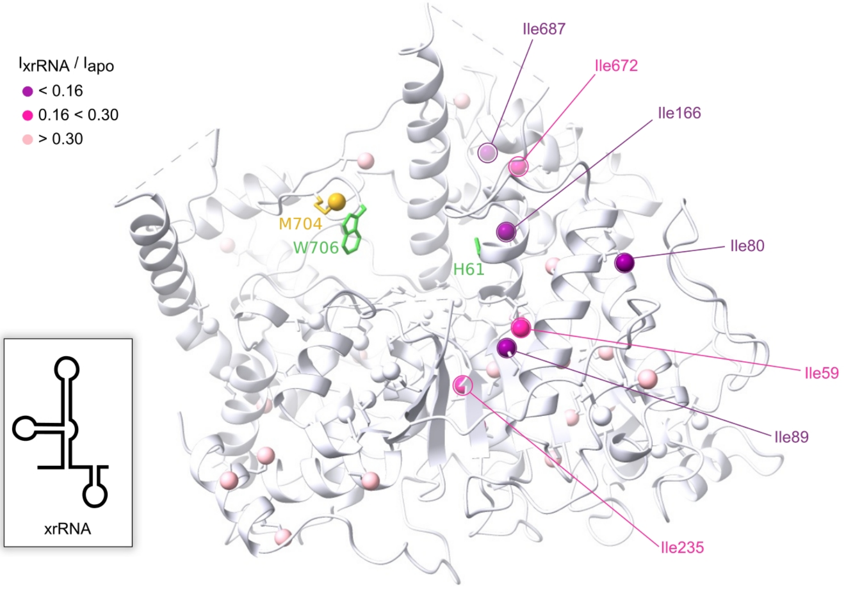

Supplementary Fig. 18 | Intensity changes in Xrn2 Ileδ$_1$-methyl groups upon addition of xrRNA

The addition of xrRNA to Xrn2 results in a significant overall decrease of the signal intensities, presumably due to an increase in the rotational correlation time and to the introduction of a large number of protons from the xrRNA. The decrease is particularly strong between the α3-helix and the active site.

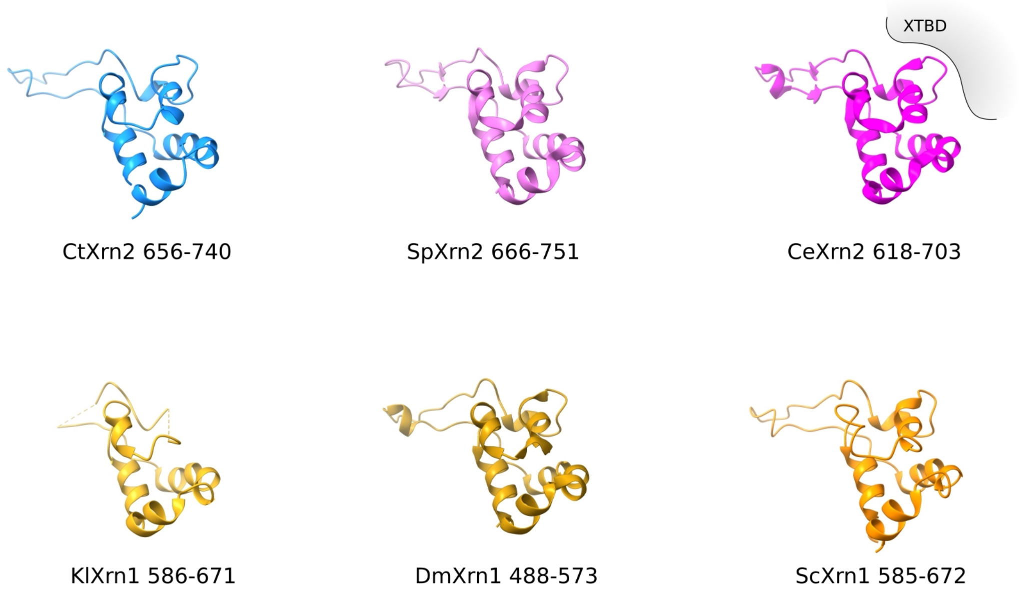

Supplementary Fig. 19 | The PBS (PAXT-1 – binding segment) of CtXrn2 (blue) is folded

For clarity, only the PBS is visualized. For comparison, the PBS from *S. pombe* Xrn2 (orchid, PDB 3FQD (2)), *C. elegans* Xrn2 (magenta, with the XTBD binding site indicated, PDB 5FIR (5)), *K. lactis* Xrn1 (yellow, PDB 3PIE (6)), *D. melanogaster* Xrn1 (gold, PDB 2Y35 (4)) and *S. cerevisiae* Xrn1 (orange, PDB 6Q8Y (3)) are shown.

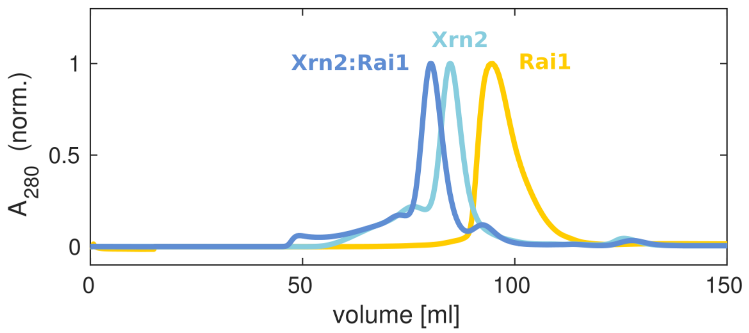

Supplementary Fig. 20 | Xrn2 and Rai1 from *Chaetomium thermophilum* form a stable complex

Shown are three exemplary size exclusion chromatography (SEC) runs of Rai1 (yellow), Xrn2 (light blue) and the Xrn2:Rai1 complex (blue). Xrn2 and Rai1 form a complex that elutes earlier than Xrn2 or Rai1 alone.

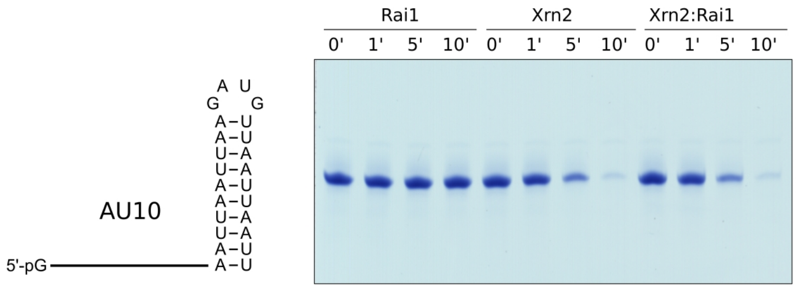

Supplementary Fig. 21 | CtRai1 does not degrade 5' monophosphorylated RNA and does not enhance the exoribonucleolytic activity of CtXrn2

A monophosphorylated AU10 RNA (12 µM) is not degraded by Rai1 (10 nM) (lanes 1 to 4). This RNA is, however, degraded readily by Xrn2 (10nM; lanes 5-8). The rate of RNA degradation is not affected by the addition of Rai1 (lanes 9 to 12). This implies that Rai1 does not influence CtXrn2 activity in *C. thermophilum*, in contrast to what was observed for the *S. pombe* proteins (2). These experiments were performed independently with three different RNA substrates.

Supplementary Table 1 | Protein constructs used in this study

Construct and residue boundaries	Tag and cleavage site	Internal reference
CtXrn2 1-875	C-His6	#1683
CtXrn2 1-875 Δ263-300	C-His6	#1821
CtXrn2 265-293	N-His6-GB1-TEV	#1820
CtXrn2 1-875 A5F	C-His6	#2331
CtXrn2 1-875 Δ263-300 N12C A5F	C-His6	#2316
CtXrn2 422-571	N-His6-TEV	#1764
CtXrn2 1-875 Δ263-300 N12C	C-His6	#1913
CtXrn2 1-875 Δ263-300 R8C	C-His6	#1982
CtXrn2 1-875 Δ263-300 R362C	C-His6	#1967
CtXrn2 1-875 Δ263-300 V416C	C-His6	#1955

Supplementary Table 2 | Constructs used for the Xrn2 resonance assignments by a mutational approach (11–13)

**Construct**	**Tag**	**Internal database reference**	**Assigned (313 K)**	**Assigned (313 K, pAp)**
CtXrn2 1-875 I3M	C-His6	#1985		x
CtXrn2 1-875 Δ435-568 I17V	C-His6	#1854		x
CtXrn2 1-875 Δ435-568 I18V	C-His6	#1831		x
CtXrn2 1-875 Δ435-568 I27V	C-His6	#1929	x	x
CtXrn2 1-875 Δ435-568 I35V	C-His6	#1924	x	x
CtXrn2 1-875 I59V	C-His6	#1810	x	x
CtXrn2 1-875 Δ435-568 I80V	C-His6	#1920	x	x
CtXrn2 1-875 Δ435-568 I82V	C-His6	#1921	x	
CtXrn2 1-875 Δ435-568 I89V	C-His6	#1856	x	x
CtXrn2 1-875 Δ435-568 I98V	C-His6	#1875	x	x
CtXrn2 1-875 Δ435-568 I101V	C-His6	#1925	x	x
CtXrn2 1-875 Δ435-568 I166V	C-His6	#1825	x	x
CtXrn2 1-875 Δ435-568 I175V	C-His6	#1922	x	x
CtXrn2 1-875 Δ435-568 I200V	C-His6	#1926	x	x
CtXrn2 1-875 Δ435-568 I201V	C-His6	#1876	x	
CtXrn2 1-875 Δ435-568 I214V	C-His6	#1894	x	x
CtXrn2 1-875 Δ435-568 I218V	C-His6	#1940	x	x
CtXrn2 1-875 Δ435-568 I235V	C-His6	#1927	x	x
CtXrn2 1-875 Δ435-568 I243V	C-His6	#1947		x
CtXrn2 1-875 Δ435-568 I308V	C-His6	#1877		x
CtXrn2 1-875 Δ435-568 I314V	C-His6	#1937	x	x
CtXrn2 1-875 Δ435-568 I338V	C-His6	#1928	x	x
CtXrn2 1-875 Δ435-568 I361V	C-His6	#1855		x
CtXrn2 1-875 Δ435-568 I366V	C-His6	#1862		x
CtXrn2 1-875 Δ435-568 I372V	C-His6	#1860		x
CtXrn2 1-875 Δ435-568 I379V	C-His6	#1930	x	x
CtXrn2 1-875 Δ435-568 I398V	C-His6	#1948	x	x
CtXrn2 1-875 Δ435-568 I409V	C-His6	#1826		x
CtXrn2 1-875 Δ435-568 (ΔI467 ΔI475 ΔI504 ΔI544)	C-His6	#1823	x	x
CtXrn2 1-875 Δ435-568 I595V	C-His6	#1949	x	x
CtXrn2 1-875 Δ435-568 I646V	C-His6	#1859	x	x
CtXrn2 1-875 Δ435-568 I653V	C-His6	#1878	x	x
CtXrn2 1-875 Δ435-568 I672V	C-His6	#1879	x	x
CtXrn2 1-875 Δ435-568 I687V	C-His6	#1931	x	x
CtXrn2 1-875 Δ435-568 I688V	C-His6	#1941	x	
CtXrn2 1-875 I697V	C-His6	#1812	x	
CtXrn2 1-875 Δ435-568 I715V	C-His6	#1832		

CtXrn2 1-875 Δ435-568 I748V	C-His6	#1938	(x)	
CtXrn2 1-875 Δ435-568 I759V	C-His6	#1932	(x)	
CtXrn2 1-875 Δ435-568 I790V	C-His6	#1942	x	
CtXrn2 1-875 Δ435-568 I817V	C-His6	#1880	(x)	
CtXrn2 1-875 Δ435-568 I822V	C-His6	#1950	x	x
CtXrn2 1-875 Δ435-568 I850V	C-His6	#1939	x	x
CtXrn2 1-875 Δ435-568 I852V	C-His6	#1951	x	x
CtXrn2 1-875 Δ435-568 I853V	C-His6	#1827	x	x
CtXrn2 1-875 Δ263-300 M704C	C-His6	#1956	x	x
			Ile: 35 assigned, 3 assigned as cluster<br>Met: 1 assigned	

Supplementary Table 3 | Rates of RNA degradation by Xrn2

RNA	Construct	Temperature [K]	Degradation rate [s^{-1}]	Figure
**AU10**	Xrn2 1-875 ΔZnF N12C	303	64.14 ± 12.25	S27
**AU10**	Xrn2 1-875 ΔZnF N12C	305.5	67.38 ± 30.22	S27
**AU10**	Xrn2 1-875 ΔZnF N12C	308	81.71 ± 32.81	S27
**AU10**	Xrn2 1-875 ΔZnF N12C	310.5	95.71 ± 36.18	S27
**AU10**	Xrn2 1-875 ΔZnF N12C	313	127.07 ± 38.53	5, S27
**AU10**	Xrn2 1-875 A5F	313	107.89 ± 12.20	5
**AU10**	Xrn2 1-875 ΔZnF N12C A5F	313	93.08 ± 2.40	5
**AU10**	Xrn2 1-875	313	162.83 ± 11.70	1, 5
**AU10**	Xrn2 1-875 ΔZnF	313	168.25 ± 20.38	1
**GC8**	Xrn2 1-875	313	35.14 ± 4.07	1
**GC8**	Xrn2 1-875 ΔZnF	313	36.69 ± 2.08	1
**GC10**	Xrn2 1-875	313	12.00 ± 2.96	1
**GC10**	Xrn2 1-875 ΔZnF	313	10.10± 0.33	1
**GC12**	Xrn2 1-875 ΔZnF N12C	303	1.99 ± 0.33	S27
**GC12**	Xrn2 1-875 ΔZnF N12C	305.5	2.47 ± 0.50	S27
**GC12**	Xrn2 1-875 ΔZnF N12C	308	3.08 ± 0.70	S27
**GC12**	Xrn2 1-875 ΔZnF N12C	310.5	3.57 ± 0.56	S27
**GC12**	Xrn2 1-875 ΔZnF N12C	313	3.83 ± 0.81	5, S27
**GC12**	Xrn2 1-875 A5F	313	3.31 ± 0.64	5
**GC12**	Xrn2 1-875 ΔZnF N12C A5F	313	2.10 ± 0.06	5
**GC12**	Xrn2 1-875	313	6.35 ± 0.86	1, 5
**GC12**	Xrn2 1-875 ΔZnF	313	4.40 ± 0.39	1
**GC14**	Xrn2 1-875	313	7.35 ± 1.10	1
**GC14**	Xrn2 1-875 ΔZnF	313	4.21 ± 0.07	1

Supplementary Table 4 | RNA sequences used in this study

**RNA**	**IVT primer sequence**	**RNA sequence**	**Internal RNA primer reference**
5-mer RNA	5’-CTTCTTTCTTCCCTTCCTTCCTCTCACTCCTATAGTGAGTCGTATTACG-3’	5’-GGAGUGAGAGGAAGGAAGGGAAGAAAGAAG-3’ (underlined = after RNase A cleavage)	8
10-mer RNA	5’-CTTCTTTCTTCCCTTCCTTCACTCTCCTCCTATAGTGAGTCGTATTACG-3’	5’-GGAGGAGAGUGAAGGAAGGGAAGAAAGAAG-3’ (underlined = after RNase A cleavage)	9
AU10	5’-AATTAATTAACATCTTAATTAATTATCTCCTTATTCTAATTCCTATAGTGAGTCGTATTACG-3’	5’-GGAAUUAGAAUAAGGAGAUAAUUAAUUAAGAUGUUAAUUAAUU-3’	110
GC8	5’-GGCCGGCCTTGCGGCCGGCCATCTCCTTATTCTAATTCCTATAGTGAGTCGTATTACG-3’	5’-GGAAUUAGAAUAAGGAGAUGGCCGGCCGCAAGGCCGGCC-3’	187
GC10	5’-CCGGCCGGCCTTGCGGCCGGCCGGATCTCCTTATTCTAATTCCTATAGTGAGTCGTATTACG-3’	5’-GGAAUUAGAAUAAGGAGAUCCGGCCGGCCGCAAGGCCGGCCGG-3’	111
GC12	5’-CCCCGGCCGGCCTTGCGGCCGGCCGGGGATCTCCTTATTCTAATTCCTATAGTGAGTCGTATTACG-3’	5’-GGAAUUAGAAUAAGGAGAUCCCCGGCCGGCCGCAAGGCCGGCCGGGG-3’	151
GC14	5’-CCCCGGCCGGCCCCTTGCGGGGCCGGCCGGGGATCTCCTTATTCTAATTCCTATAGTGAGTCGTATTACG-3’	5’-GGAAUUAGAAUAAGGAGAUCCCCGGCCGGCCCCGCAAGGGGCCGGCCGGGG-3’	152
ZIKV xrRNA	5’-CCCACTTTCGTGGGGGGGTTACAGGCTGCACAGCTTTCCCCAAACTGTGGCGACTTTCGCCGGCCTGACAACACTAAAATTGGTGCTTACAACACTCCCTATAGTGAGTCGTATTACG-3’	5’-GGGAGUGUUGUAAGCACCAAUUUUAGUGUUGUCAGGCCGGCGAAAGUCGCCACAGUUUGGGGAAAGCUGUGCAGCCUGUAACCCCCCCACGAAAGUGGG-3’	186

Supplementary Table 5 | Point mutants of the Xrn2 ΔZnF N12C construct

Mutant	Location	Comment
P4G	N-terminal helix	no expression
P4K	N-terminal helix	poor labeling
A5G	N-terminal helix	poor labeling, aggregation at 313 K
A5M	N-terminal helix	similar dispersions
A5F	*N-terminal helix*	*significantly changed dispersions*
A5V	N-terminal helix	poor labeling
A6G	N-terminal helix	aggregation at 303 K
A6M	N-terminal helix	aggregation at 313 K, changed dispersions
A6F	N-terminal helix	poor labeling
A6V	N-terminal helix	poor labeling
F7I	N-terminal helix	no expression
F7Y	N-terminal helix	CPMG profile similar to WT
R8K	N-terminal helix	poor labeling
W9F	N-terminal helix	poor labeling
L10I	N-terminal helix	poor labeling
Y14F	N-terminal helix	poor labeling
K16M	Next to helix	poor labeling
A240G	Active center	CPMG profile similar to WT
A240V	Active center	poor labeling
A240M	Active center	poor labeling
I853L	C-terminal end	CPMG profile similar to WT
A860E	C-terminal end	poor labeling

Supplementary Table 6 | Fit parameters for RD data

^{13}C data: fit parameters

**MC simulations**[1]							
			Ile59	Ile89	Ile235	Ile850	Ile853
	p_{GS}	k_{ex} [s^{-1}]	$\|\Delta\omega_C\|$ [ppm]	$\|\Delta\omega_C\|$ [ppm]	$\|\Delta\omega_C\|$ [ppm]	$\|\Delta\omega_C\|$ [ppm]	$\|\Delta\omega_C\|$ [ppm]
**CH_3**	0.829 ± 0.062	722 ± 63	0.26 ± 0.04	0.25 ± 0.03	0.51 ± 0.07	0.16 ± 0.02	0.68 ± 0.09
**Optimal solution**[2]							
			Ile59	Ile89	Ile235	Ile850	Ile853
	p_{GS}	k_{ex} [s^{-1}]	$\|\Delta\omega_C\|$ [ppm]	$\|\Delta\omega_C\|$ [ppm]	$\|\Delta\omega_C\|$ [ppm]	$\|\Delta\omega_C\|$ [ppm]	$\|\Delta\omega_C\|$ [ppm]
**CH_3**	**0.819 ± 0.062**	**710 ± 63**	**0.25 ± 0.04**	**0.24 ± 0.03**	**0.47 ± 0.07**	**0.15 ± 0.02**	**0.64 ± 0.09**

^{19}F data: fit parameters

**MC simulations**[1]											
	303 K		305.5 K		308 K		310.5		313 K		
	p_{GS}	k_{ex} [s^{-1}]	p_{GS}	k_{ex} [s^{-1}]	p_{GS}	k_{ex} [s^{-1}]	p_{GS}	k_{ex} [s^{-1}]	p_{GS}	k_{ex} [s^{-1}]	$\|\Delta\omega\|$ [ppm]
**CPMG**	0.702 ± 0.115	296 ± 145	0.723 ± 0.086	297 ± 136	0.763 ± 0.097	423 ± 161	0.794 ± 0.117	646 ± 178	0.809 ± 0.121	780 ± 189	0.216 ± 0.048
**$R_{1\rho}$**	0.663 ± 0.051	501 ± 78	0.560 ± 0.068	570 ± 95	0.575 ± 0.050	608 ± 31	0.600 ± 0.056	654 ± 47	0.598 ± 0.063	898 ± 144	0.153 ± 0.006
**CPMG + $R_{1\rho}$**	0.651 ± 0.051	491 ± 67	0.656 ± 0.048	574 ± 74	0.588 ± 0.044	612 ± 30	0.625 ± 0.047	681 ± 43	0.628 ± 0.057	871 ± 108	0.150 ± 0.006
**Optimal solution**[2]											
	303 K		305.5 K		308 K		310.5		313 K		
	p_{GS}	k_{ex} [s^{-1}]	p_{GS}	k_{ex} [s^{-1}]	p_{GS}	k_{ex} [s^{-1}]	p_{GS}	k_{ex} [s^{-1}]	p_{GS}	k_{ex} [s^{-1}]	$\|\Delta\omega\|$ [ppm]
**CPMG**	0.62 ± 0.115	459 ± 145	0.59 ± 0.086	494 ± 136	0.56 ± 0.097	617± 161	0.50 ± 0.117	826 ± 178	0.50 ± 0.121	987 ± 189	0.153 ± 0.048
**$R_{1\rho}$**	0.64 ± 0.051	469 ± 78	0.50 ± 0.068	521± 95	0.50 ± 0.050	612± 31	0.55± 0.056	648 ± 47	0.50 ± 0.063	876 ± 144	0.147 ± 0.006
**CPMG + $R_{1\rho}$**	**0.62 ± 0.051**	**445 ± 67**	**0.56± 0.048**	**508 ± 74**	**0.50 ± 0.044**	**627± 30**	**0.55 ± 0.047**	**686 ± 43**	**0.50 ± 0.057**	**913 ± 108**	**0.149 ± 0.006**

^{19}F + ^{13}C data: fit parameters

**MC simulations**[1]								
			Ile59	Ile89	Ile235	Ile850	Ile853	^{19}F
	p_{GS}	k_{ex} [s^{-1}]	$\|\Delta\omega_C\|$ [ppm]	$\|\Delta\omega_C\|$ [ppm]	$\|\Delta\omega_C\|$ [ppm]	$\|\Delta\omega_C\|$ [ppm]	$\|\Delta\omega_C\|$ [ppm]	$\|\Delta\omega_F\|$ [ppm]
**CH_3+ ^{19}F**	0.707 ± 0.065	824 ± 112	0.20 ± 0.05	0.19 ± 0.05	0.40 ± 0.03	0.12 ± 0.03	0.52 ± 0.04	0.17 ± 0.01
**Optimal solution**[2]								
			Ile59	Ile89	Ile235	Ile850	Ile853	^{19}F
	p_{GS}	k_{ex} [s^{-1}]	$\|\Delta\omega_C\|$ [ppm]	$\|\Delta\omega_C\|$ [ppm]	$\|\Delta\omega_C\|$ [ppm]	$\|\Delta\omega_C\|$ [ppm]	$\|\Delta\omega_C\|$ [ppm]	$\|\Delta\omega_F\|$ [ppm]
**CH_3+ ^{19}F**	**0.50 ± 0.065**	**864 ± 112**	**0.19 ± 0.05**	**0.18 ± 0.05**	**0.34 ± 0.03**	**0.11 ± 0.03**	**0.45 ± 0.04**	**0.15 ± 0.01**

[1] Given are the average value and the standard deviation for the fitting parameters, based on 500 MC cycles (Supplementary Fig.13,18 and 19).

[2] The optimal solution is the solution with the lowest reduced χ^2 value. Note that this is not necessarily the same as the average value that is obtained from the MC derived fitting parameters. In addition, the average MC values are not necessarily a solution to the fitting problem, as the fitting parameters depend on another in a non-linear manner. The indicated error correspond to MC errors.

Supplementary Table 7 | Crystallography: Data collection and refinement statistics

	CtXrn2:Mg^{2+} (PDB 7OPK)
**Data collection**	
Space group	$P\ 3_1\ 2\ 1$
Cell dimensions	
a, b, c (Å)	198.78, 198.78, 69.14
α, β, γ (º)	90, 90, 120
Resolution (Å)	47.75 – 3.00 (3.11 – 3.00)[a]
R_{pim}	0.068 (0.455)
R_{meas}	0.309 (2.037)
I/σ(I)	14.74 (2.17)
$CC_{1/2}$	0.996 (0.786)
Completeness (%)	99.85 (99.94)
Redundancy	20.4 (19.9)
**Refinement**	
Resolution (Å)	47.75 – 3.00 (3.11 – 3.00)[a]
No. reflections	31580
R_{work} / R_{free} (%)	18.3 / 22.4
No. atoms	
Protein	5306
Ion (Mg^{2+})	2
Water	1
Bfactors	64.64
Protein	64.65
Ion (Mg^{2+})	49.14
Water	42.53
R.m.s. deviations	
Bond lengths (Å)	0.003
Bond angles (º)	0.57

One crystal was used for the structure

[a] Values in parentheses show data for the highest resolution shell

Supplementary Table 8 | Structural statistics for the NMR solution structure of the zinc finger

	CtXrn2 Zincfinger:Zn^{2+} (PDB 7PVM)
**Completeness of resonance assignments**	
Backbone	91.3 %
Side chain	70.1 %
**Conformationally restricting restraints**	
Distance restraints	
Total	380
Short range (|i-j| <= 5)	227 (59.7 %)
Medium range (1 < |i-j| < 5)	79 (20.8 %)
Long range (|i-j| ≥ 5)	74 (19.7 %)
No. of restraints per residue	12.3
No. of long-range restraints per residue	2.4
Distance restraint violations	
Number of violations > 0.1 Å	0
RMS	0.0036 ± 0.0005 Å
Ramachandran statistics (%)	
Most favored regions	74.0
Additionally allowed regions	25.8
Generously allowed regions	0.2
Disallowed regions	0.0
Model quality	
RMSD backbone atoms (Å) (residues 8-23)	0.03 ± 0.01 Å
RMSD heavy atoms (Å) (residues 8-23)	0.57 ± 0.15 Å

Supplementary references

1. M. Jinek, S. M. Coyle, J. A. Doudna, Coupled 5′ Nucleotide Recognition and Processivity in Xrn1-Mediated mRNA Decay. *Mol. Cell* **41**, 600–608 (2011).

2. S. Xiang, *et al.*, Structure and function of the 5′→3′ exoribonuclease Rat1 and its activating partner Rai1. *Nature* **458**, 784–788 (2009).

3. P. Tesina, *et al.*, Structure of the 80S ribosome–Xrn1 nuclease complex. *Nat. Struct. Mol. Biol.* **26**, 275–280 (2019).

4. M. Jinek, S. M. Coyle, J. A. Doudna, Coupled 5′ Nucleotide Recognition and Processivity in Xrn1-Mediated mRNA Decay. *Mol. Cell* **41**, 600–608 (2011).

5. H. Richter, I. Katic, H. Gut, H. Großhans, Structural basis and function of XRN2 binding by XTB domains. *Nat. Struct. Mol. Biol.* **23**, 164–171 (2016).

6. J. H. Chang, S. Xiang, K. Xiang, J. L. Manley, L. Tong, Structural and biochemical studies of the 5′ → 3′ exoribonuclease Xrn1. *Nat. Struct. Mol. Biol.* **18**, 270–276 (2011).

7. S. B. Miller, F. Z. Yildiz, J. A. Lo, B. Wang, V. M. D'Souza, A structure-based mechanism for tRNA and retroviral RNA remodelling during primer annealing. *Nature* **515**, 591–595 (2014).

8. A. Dey, D. York, A. Smalls-Mantey, M. F. Summers, Composition and Sequence-Dependent Binding of RNA to the Nucleocapsid Protein of Moloney Murine Leukemia Virus ,. *Biochemistry* **44**, 3735–3744 (2005).

9. G. K. Amarasinghe, *et al.*, NMR structure of the HIV-1 nucleocapsid protein bound to stem-loop SL2 of the Ψ-RNA packaging signal. implications for genome recognition 1 1Edited by P. Wright. *J. Mol. Biol.* **301**, 491–511 (2000).

10. Y. Gao, *et al.*, Structural basis for guide RNA trimming by RNase D ribonuclease in *Trypanosoma brucei*. *Nucleic Acids Res.* **49**, 568–583 (2021).

11. R. Sprangers, L. E. Kay, Quantitative dynamics and binding studies of the 20S proteasome by NMR. *Nature* **445**, 618–622 (2007).

12. R. Sprangers, A. Gribun, P. M. Hwang, W. A. Houry, L. E. Kay, Quantitative NMR spectroscopy of supramolecular complexes: dynamic side pores in ClpP are important for product release. *Proc. Natl. Acad. Sci. U. S. A.* **102**, 16678–16683 (2005).

13. S. Schütz, R. Sprangers, Methyl TROSY spectroscopy: A versatile NMR approach to study challenging biological systems. *Prog. Nucl. Magn. Reson. Spectrosc.*, S0079656519300470 (2019).
