# Supplementary material for: Observation of conformational changes that underlie the catalytic cycle of Xrn2
Source: Nat Chem Biol. 2022 Aug 25;18(10):1152–60. doi: 10.1038/s41589-022-01111-6 (PMC9512700; doi:10.1038/s41589-022-01111-6)
Supplement: Supplementary file 2 — Reporting Summary [file 41589_2022_1111_MOESM2_ESM.pdf]

nature research

Corresponding author(s): Remco Sprangers

Last updated by author(s): Jun 29, 2022

Reporting Summary

Nature Research wishes to improve the reproducibility of the work that we publish. This form provides structure for consistency and transparency in reporting. For further information on Nature Research policies, see our Editorial Policies and the Editorial Policy Checklist.

Statistics

For all statistical analyses, confirm that the following items are present in the figure legend, table legend, main text, or Methods section.

n/a	Confirmed	
☐	☒	The exact sample size (n) for each experimental group/condition, given as a discrete number and unit of measurement
☐	☒	A statement on whether measurements were taken from distinct samples or whether the same sample was measured repeatedly
☒	☐	The statistical test(s) used AND whether they are one- or two-sided<br>*Only common tests should be described solely by name; describe more complex techniques in the Methods section.*
☒	☐	A description of all covariates tested
☒	☐	A description of any assumptions or corrections, such as tests of normality and adjustment for multiple comparisons
☐	☒	A full description of the statistical parameters including central tendency (e.g. means) or other basic estimates (e.g. regression coefficient) AND variation (e.g. standard deviation) or associated estimates of uncertainty (e.g. confidence intervals)
☒	☐	For null hypothesis testing, the test statistic (e.g. F, t, r) with confidence intervals, effect sizes, degrees of freedom and P value noted<br>*Give P values as exact values whenever suitable.*
☒	☐	For Bayesian analysis, information on the choice of priors and Markov chain Monte Carlo settings
☒	☐	For hierarchical and complex designs, identification of the appropriate level for tests and full reporting of outcomes
☒	☐	Estimates of effect sizes (e.g. Cohen's d, Pearson's r), indicating how they were calculated

Our web collection on statistics for biologists contains articles on many of the points above.

Software and code

Policy information about availability of computer code

Data collection	NMR data collection: Topspin 4<br>Xray data collection: PXII beamline software at the Swiss Light Source
Data analysis	NMR data processing: Topspin 4 and NMRPipe/ NMRDraw version 9.6<br>NMR data analysis: NMRFAM-SPARKY 1.2 powered by Sparky 3.115, CARA 1.9.1.8a2 and NMRViewJ 9.2.0-b24<br>NMR structure calculations: Cyana 3.98.13<br>X-ray structure calculations: XDS 2021, CCP4 7.1, Phaser 2.8.3, Coot 0.9.6 and Phenix 1.18.2-3874<br>Data fitting: Matlab R2021a<br>Visualization: ChimeraX 1.1<br>Sequence analysis: ClustalX 2.1

For manuscripts utilizing custom algorithms or software that are central to the research but not yet described in published literature, software must be made available to editors and reviewers. We strongly encourage code deposition in a community repository (e.g. GitHub). See the Nature Research guidelines for submitting code & software for further information.

Data

Policy information about availability of data

All manuscripts must include a data availability statement. This statement should provide the following information, where applicable:

- Accession codes, unique identifiers, or web links for publicly available datasets
- A list of figures that have associated raw data
- A description of any restrictions on data availability

The structure factors and coordinates for the Xrn2 core protein have been deposited in the Protein Data Bank under accession code 7OPK. The assignments for the Xrn2 zinc finger have been deposited in the Biological Magnetic Resonance Bank under accession number 50997. The atomic coordinates for the Xrn2 zinc finger have been deposited in the Protein Data Bank under accession code 7PVM. All other relevant data are available in the source data provided with this manuscript and are also available upon request to the corresponding author.

Field-specific reporting

Please select the one below that is the best fit for your research. If you are not sure, read the appropriate sections before making your selection.

☒ Life sciences ☐ Behavioural & social sciences ☐ Ecological, evolutionary & environmental sciences

For a reference copy of the document with all sections, see nature.com/documents/nr-reporting-summary-flat.pdf

Life sciences study design

All studies must disclose on these points even when the disclosure is negative.

Sample size	The exact sample size and the number of replicate experiments have been described in the figure legends and methods.
Data exclusions	No data (points) have been omitted.
Replication	All data were recorded as at least fully independent duplicates. All attempts at replication were successful.
Randomization	Randomization has no relevance in this study.
Blinding	Blinding has no relevance in this study.

Reporting for specific materials, systems and methods

We require information from authors about some types of materials, experimental systems and methods used in many studies. Here, indicate whether each material, system or method listed is relevant to your study. If you are not sure if a list item applies to your research, read the appropriate section before selecting a response.

Materials & experimental systems

n/a	Involved in the study
☒	☐ Antibodies
☒	☐ Eukaryotic cell lines
☒	☐ Palaeontology and archaeology
☒	☐ Animals and other organisms
☒	☐ Human research participants
☒	☐ Clinical data
☒	☐ Dual use research of concern

Methods

n/a	Involved in the study
☒	☐ ChIP-seq
☒	☐ Flow cytometry
☒	☐ MRI-based neuroimaging
